# Targeting calcium-crosslinked alginate hydrogel in a living subject using a synthetic anion receptor

**DOI:** 10.1039/d6sc05110c

**Published:** 2026-08-25

**Authors:** Hunter B. D. Cheney, Rananjaya S. Gamage, Jon Chiaramonte, Catherine G. Price, Colin Davidson, Donny Hanjaya-Putra, Bradley D. Smith

**Affiliations:** a Department of Chemistry and Biochemistry, University of Notre Dame Notre Dame IN 46556 USA smith.115@nd.edu; b Bioengineering Program, Department of Aerospace and Mechanical Engineering, University of Notre Dame Notre Dame IN 46556 USA

## Abstract

Alginate hydrogels are widely utilized in different medical applications as drug excipients, wound dressings, and tissue engineering scaffolds. However, selective delivery of molecular payload to unmodified alginate hydrogels in complex biological media remains a significant challenge. This study presents a novel supramolecular approach for targeting unmodified alginate hydrogels using synthetic zinc(ii) bis(2,2′-dipicolylamine) (ZnBDPA) coordination complexes. Recognizing the polyanionic nature of alginate, we demonstrate that ZnBDPA receptors exhibit high binding affinity for the carboxylate groups on an alginate polymer backbone. Solution-state titration and dye displacement assays confirmed strong binding of a ZnBDPA receptor to different polycarboxylates including alginate. Subsequent hydrogel loading and leakage experiments found that a fluorescent ZnBDPA receptor molecule readily transferred into calcium-crosslinked alginate microspheres and remained trapped, unlike an untargeted control dye. Moreover, release of the trapped ZnBDPA receptor could be triggered by adding pyrophosphate as a receptor binding and displacement agent. Finally, *in vivo* fluorescence imaging of a living mouse revealed that intravenously administered ZnBDPA receptor selectively targeted a subcutaneously implanted alginate microsphere. These findings establish ZnBDPA as an effective delivery vehicle for alginate hydrogel implants within a living subject, offering a versatile platform for loading and controlled release of molecular payload.

## Abstract 2

A zinc-dipicolylamine (ZnBDPA) receptor has affinity for the carboxylate groups on an unmodified alginate polymer and can target an implanted calcium-crosslinked alginate microsphere, offering a new supramolecular platform for payload delivery.

## Graphical abstract

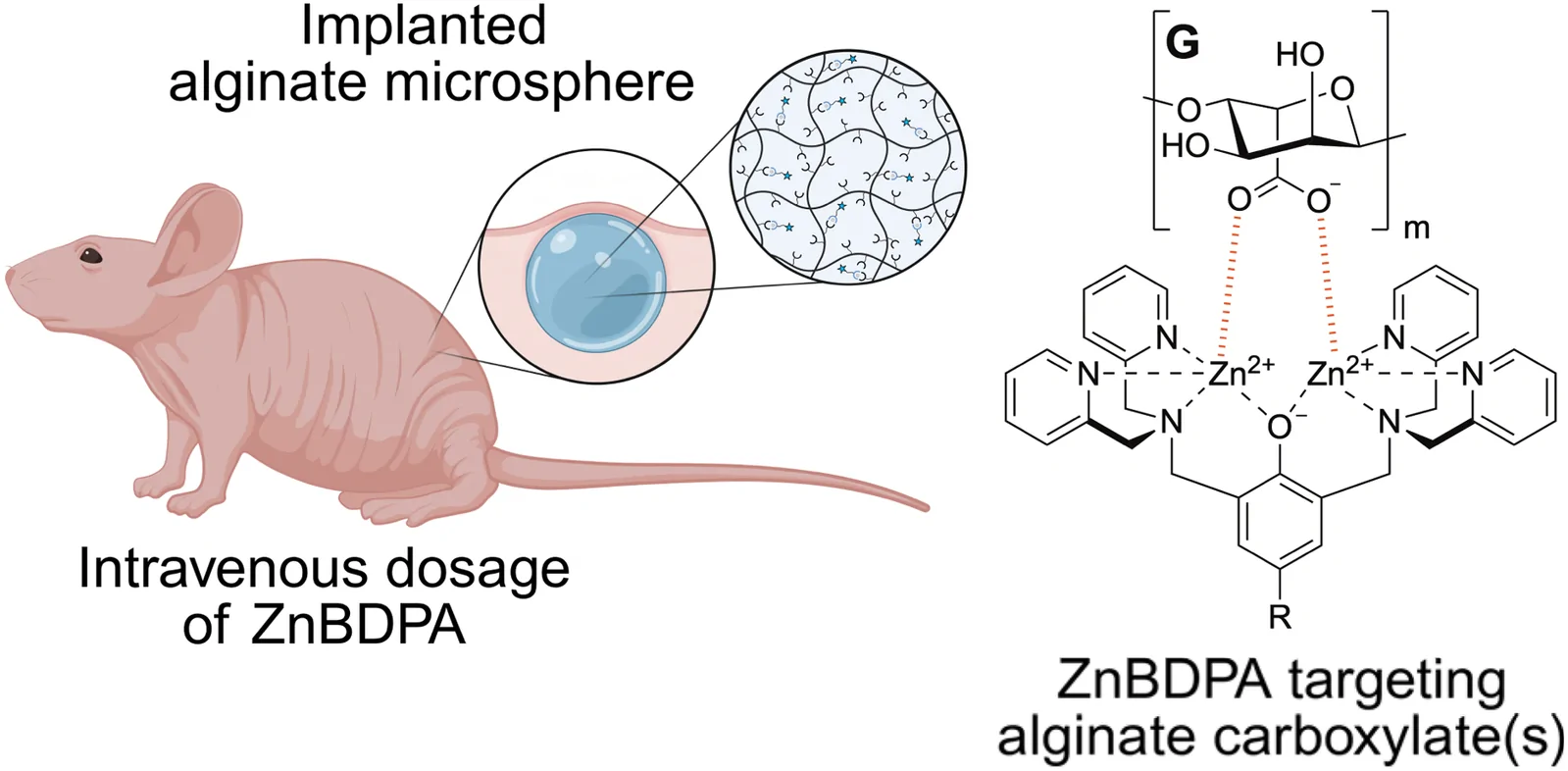

## Introduction

**Scheme 1:**
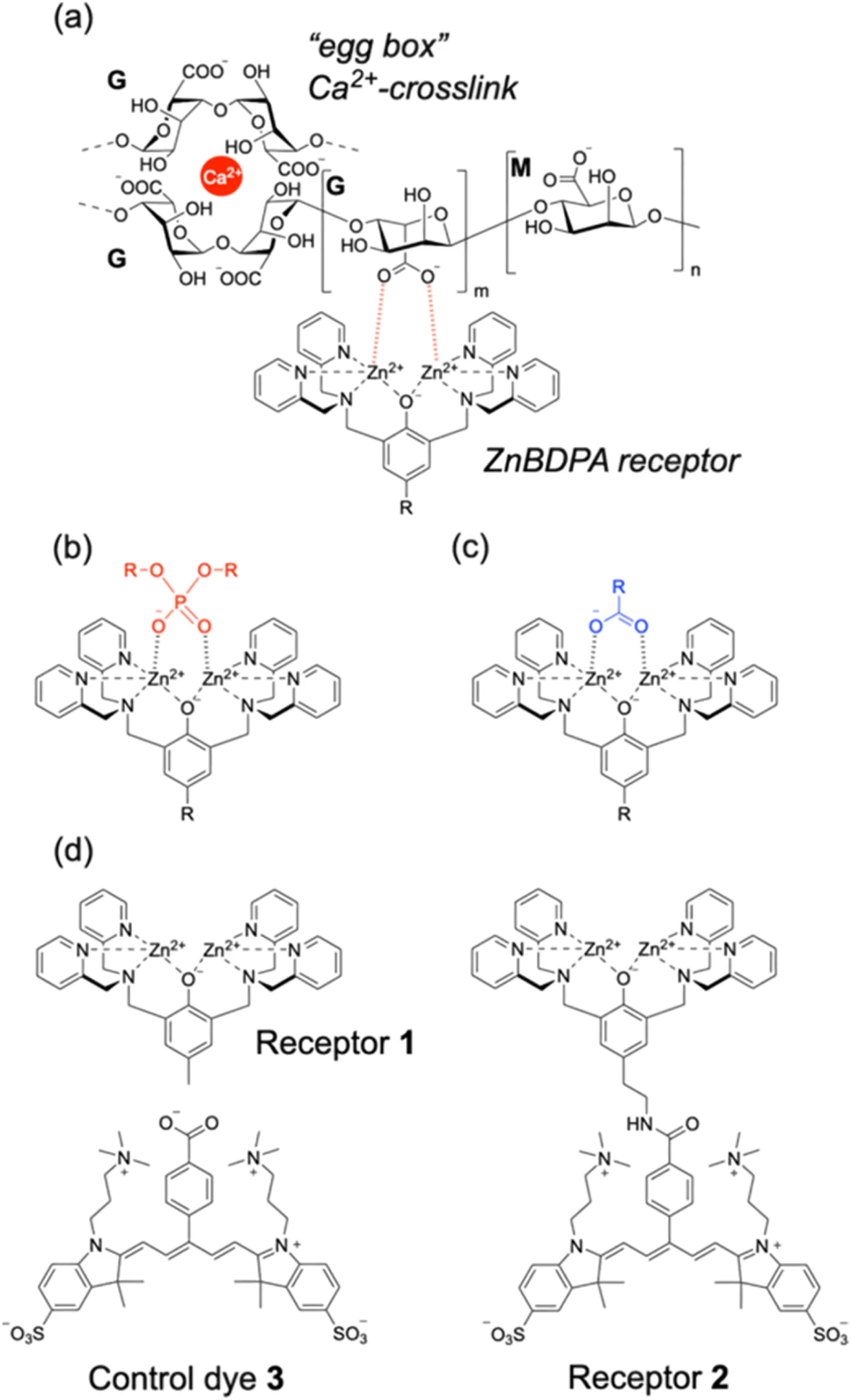
(a) Illustration of an alginate strand composed of M and G residues, the “egg-box” model for Ca^2+^-mediated cross-linking of G-blocks, and association of a ZnBDPA receptor with an alginate carboxylate group. ZnBDPA receptor association with, (b) phosphate diester, and (c) carboxylate. (d) Structures of the ZnBDPA receptors (1–2) and control dye (3) used in this study.

Commercial alginate is a long polysaccharide derived from marine brown algae and its structure is composed of α-l-guluronic acid (G) and β-d-mannuronic acid (M) monomeric residues linked through β-1,4 bonds with a common commercial molecular weight range of 33 000 to 400 000 g mol^−1^.^1^ The natural polymer sequence may occur in blocks of consecutive G-residues (G-blocks), consecutive M-residues (M-blocks) or alternating G and M-residues (GM-blocks). Cross-linked alginate readily forms hydrogels that are used extensively as food additives, drug excipients, scaffolds for cell culture, wound dressings, and as capsules for controlled release of molecular or cellular payload.^1,2^ There is an array of fabrication methods to cross-link alginate polymers and form robust hydrogels.^3^ For example, robust hydrogel microspheres are produced under very mild conditions when the water-soluble sodium salt of alginate is added dropwise into a solution containing a high concentration of divalent cations (*e.g.*, Ca^2+^). The divalent cations associate with the carboxylate units within a homopolymeric G-block and form multiple cross-links according to the “egg-box” cross-linking model (Scheme 1a).^4^ The mild cross-linking process allows the microsphere to trap various types of payload such as drugs, biopolymers, enzymes, or living cells, and many of these loaded microspheres are under active preclinical investigation as medical implants.^5–7^

The objective of this study was to develop a supramolecular method for delivering molecular payload to a Ca-crosslinked alginate microsphere implanted in a living subject. Achieving this goal would allow a Ca-crosslinked alginate microsphere to act as a depot for loading and localized controlled release. To date, this general idea has been approached by synthetically modifying a hydrogel-forming polymer (such as alginate) to permit spontaneous loading of molecular payload by two different mechanisms, one based on covalent click chemistry,^8,9^ and the other based on non-covalent supramolecular association.^10–17^ A limitation with both approaches is the requirement to synthesize and purify the modified polymer structures and the need to verify that they exhibit satisfactory biocompatibilities.^18^ We reasoned that the technological impact would be much broader if the molecular payload could be designed to non-covalently bind to the unmodified hydrogel-forming polymer. In the case of Ca-crosslinked alginate microspheres, this means payload molecules with high and selective affinity for the alginate strands within the microsphere. At present the only reported examples of molecules with alginate affinity are some recently described lectin proteins^19–21^ and DNA aptamers.^22^ We decided to approach this molecular recognition challenge from the perspective of synthetic anion receptor chemistry and treat the alginate polymer scaffold as a supramolecular target with an anionic carboxylate group on each residue.

Our previous experience with synthetic anion receptors led us to consider zinc(ii) bis(2,2′-dipicolylamine) (ZnBDPA) coordination complexes as alginate targeting units.^23^ ZnBDPA receptors are primarily used for binding anionic biomolecules that contain polyphosphate groups or anionic membrane surfaces with multiple copies of phosphate diesters (Scheme 1b).^24–26^ ZnBDPA receptors have relatively weak affinity for target molecules that contain a single carboxylate group (Scheme 1c); however, carboxylate affinity is greatly enhanced if the recognition partners have structures that permit multivalent association. For example, Hamachi and coworkers have reported that a receptor molecule with two ZnBDPA units binds tetra-aspartate (a peptide sequence containing four carboxylates) with *K*_a_ ∼10^4^ M^−1^ in pH 7.4 buffer, whereas an analogous receptor with four ZnBDPA units exhibits >1000 times higher affinity (*K*_a_ ∼10^7^ M^−1^) for a peptide sequence containing eight carboxylates.^24,27,28^ Moreover, these strong and selective peptide polycarboxylate affinities are maintained in competitive media such as phosphate buffer and cell culture.^24^

Here, we report the first study of ZnBDPA binding to free polyoxyanions, including alginate, and to the alginate strands within Ca-crosslinked alginate hydrogel microspheres (Scheme 1a). First, we describe solution-state titration experiments that measure the relative affinity of ZnBDPA receptors 1 and 2 (Scheme 1d) for monomeric and polymeric oxyanions (carboxylates and phosphates). A subsequent series of microsphere loading and leakage experiments show that fluorescent ZnBDPA receptor 2 has a much higher affinity than the control dye 3 for hydrogel microspheres composed of Ca-crosslinked alginate. Finally, *in vivo* fluorescence imaging experiments demonstrate that ZnBDPA receptor 2 can selectively target a Ca-crosslinked alginate microsphere that is implanted within a living mouse.

## Results and discussion

### Receptor synthesis and structure

Compounds 1–3 were synthesized and characterized by the methods described in the SI. The ZnBDPA unit is a derivative of tyramine and its structure contains a central oxygen atom that bridges two adjacent zinc cations to give a net +3 charge and capacity to form reversible chelated coordination complexes with a phosphate or carboxylate anion.^29^ ZnBDPA receptor 2 has an appended deep-red pentamethine cyanine fluorophore, and the unconjugated fluorophore (control dye 3) was used for comparison experiments. As summarized in Table S1, the deep-red absorption and emission properties of 2 and 3 are identical, and they are both visualized as an intense blue color. Notably, the fluorophore structure includes a distributed balance of cationic and anionic residues that provide very high water-solubility and net-zero molecular charge.^30^ While the complexation picture in Scheme 1a indicates bidentate coordination of an alginate G-carboxylate to the two Zn^2+^ cations within the ZnBDPA unit, a dynamic exchange of different coordination geometries is expected including monodentate coordination and simultaneous Zn^2+^ coordination of a proximal hydroxyl or a second alginate carboxylate.^31^

### ZnBDPA polyoxyanion binding measured by dye displacement assay

**Fig. 1:**
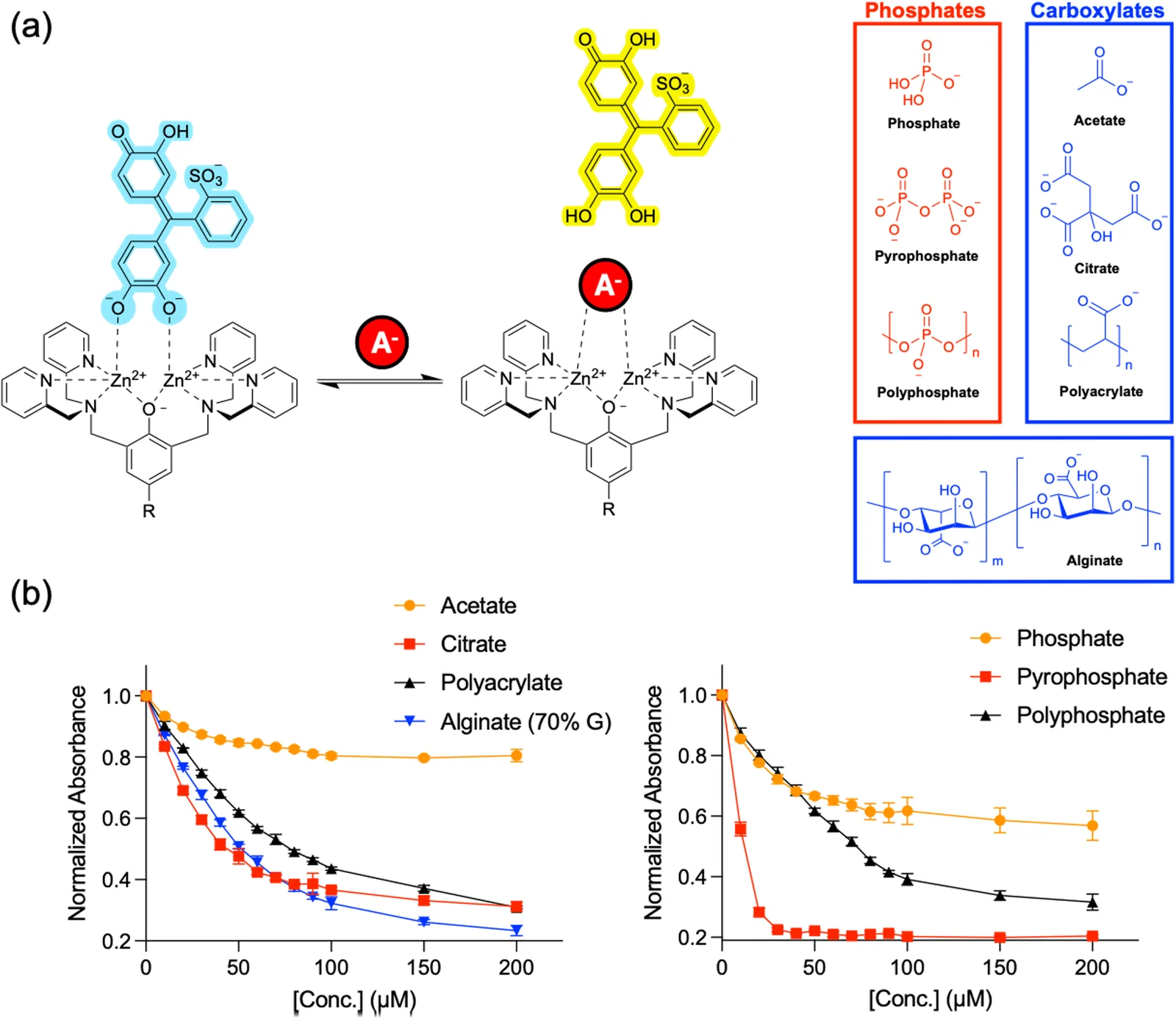
(a) Colorimetric pyrocatechol violet (PV) displacement assay and structures of the added anions, A^−^. (b) Plots showing change in absorbance at 631 nm for a binary mixture of 1 and PV (each 20 µM) in HEPES buffer (10 mM, pH 7.2) upon addition of different carboxylate (left) or phosphate anions (right). Error bars for triplicate experiments.

The first series of titration experiments employed a known dye displacement assay to assess the capacity of ZnBDPA receptor 1 to associate with different phosphate and carboxylate anions as free molecules.^32,33^ As illustrated in Fig. 1a, a binary mixture of ZnBDPA receptor 1 and pyrocatechol violet (PV) appears blue to the naked eye due to formation of a 1:PV complex with an absorption maximum at 631 nm. Subsequent titration and oxyanion binding to the Zn^2+^ cations within 1 displaces the PV dye and lowers the 631 nm absorption intensity. Shown in Fig. 1b are the results of separate titration experiments that added aliquots of different oxyanions to a binary mixture of ZnBDPA receptor 1 and PV (each 20 µM) in a solution of 10 mM HEPES buffer, pH 7.2, and measured the decrease in absorption intensity at 631 nm (Fig. S3–S4). The resulting titration isotherms indicated relative oxyanion affinity for ZnBDPA receptor 1 with the titration plots for phosphate binding showing an affinity preference for pyrophosphate > polyphosphate > phosphate, and the titration plots for the different carboxylate anions indicating a ZnBDPA 1 affinity trend of citrate > alginate ∼ polyacrylate > acetate. The relatively low ZnBDPA affinity for phosphate and acetate agrees with the literature, and the relatively high affinity for pyrophosphate and citrate is attributed to the capacity of these small polyanions to form stable multidentate chelates with the two Zn^2+^ cations within receptor 1.††Literature association constants (*K*_a_) for binding to receptor 1 are: PV = 4.45 × 10^4^ M^−1^, phosphate = 8.60 × 10^4^ M^−1^, and pyrophosphate = 1.01 × 10^7^ M^−1^ (ref. 64), and for citrate binding to a ZnBDPA receptor very similar to 1, *K*_a_ = 9.2 × 10^5^ M^−1^ (ref. 65). This current study measured the following *K*_a_ values for binding to receptor 1: alginate = 6.5 × 10^4^ M^−1^, chondroitin sulfate *B* = 1.5 × 10^5^ M^−1^, heparan sulfate = 6.0 × 10^5^ M^−1^ (see Fig. S5). The moderate affinity for the anionic polymers (*i.e.*, polyphosphate, polyacrylate, alginate) is attributed to multivalency factors such as increased electrostatic attraction and increased rates of statistical rebinding of the ZnBDPA receptor to one of the multiple monoanionic sites within the polymer.^34^ Control titration experiments with alginates composed of two quite different G/M ratios produced very similar binding isotherms (Fig. S5) indicating negligible difference in receptor 1 affinity. Interestingly, receptor 1 exhibited relatively high affinity for chondroitin sulfate and heparan sulfate which are examples of anionic glycosaminoglycans (GAGs) with polymeric structures containing a mixture of sulfate and carboxylate groups.† This binding behavior aligns with the conclusions of literature biological studies that observed retention of a ZnBDPA receptor directly injected into healthy cartilage tissue that is rich in anionic GAGs.^35,36^

### Binding of fluorescent receptor 2 to polycarboxylates

**Fig. 2:**
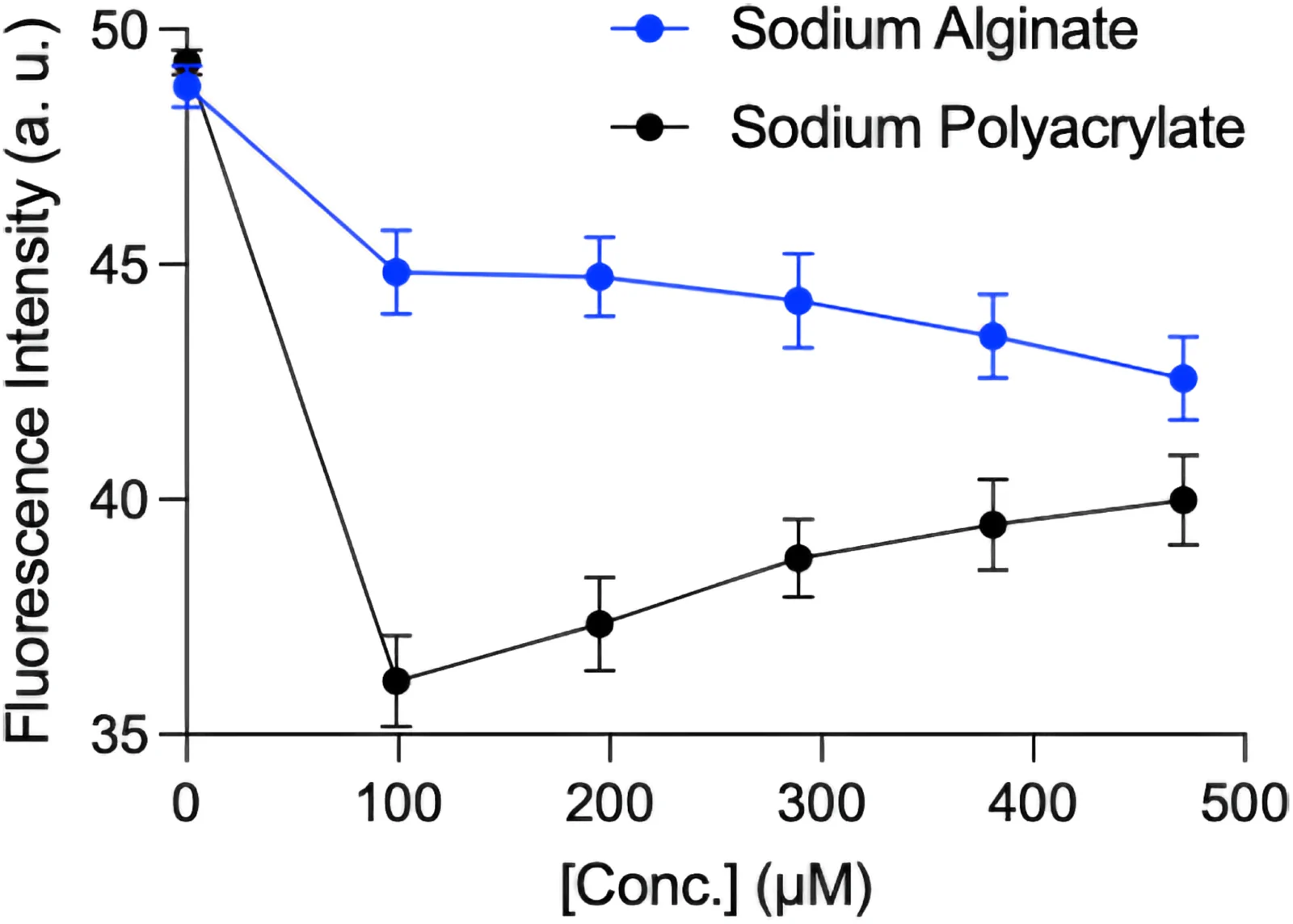
Change in fluorescence intensity at 666 nm (ex: 630 nm) for ZnBDPA receptor 2 (5 µM) in HEPES buffer (10 mM, pH 7.2) upon titration with added sodium alginate or polyacrylate (0–500 µM).

A second series of titration experiments added aliquots of polyacrylate or alginate to a solution of ZnBDPA receptor 2 (5 µM) in HEPES buffer (10 mM, pH 7.2) and observed a decrease in receptor fluorescence (Fig. 2). The polyacrylate titration plot noticeably indicates a two-phase binding process, with the first phase of polyacrylate addition producing fluorescence quenching and the later phase of polyacrylate addition producing a fluorescence rebound. This two-phase binding profile is consistent with clustering of bound and self-quenched receptor 2 during the first phase of the titration when there is high occupancy of 2 on a polyacrylate strand, and less clustering of 2 during the later phase of the titration when there is lower occupancy and greater spatial separation of bound 2.^37,38^ Clustering and quenching of cationic fluorophores upon binding to solution-state polyacrylate or alginate polyanions has been reported before.^39–41^

### Leakage from Ca-crosslinked alginate hydrogel microspheres

**Fig. 3:**
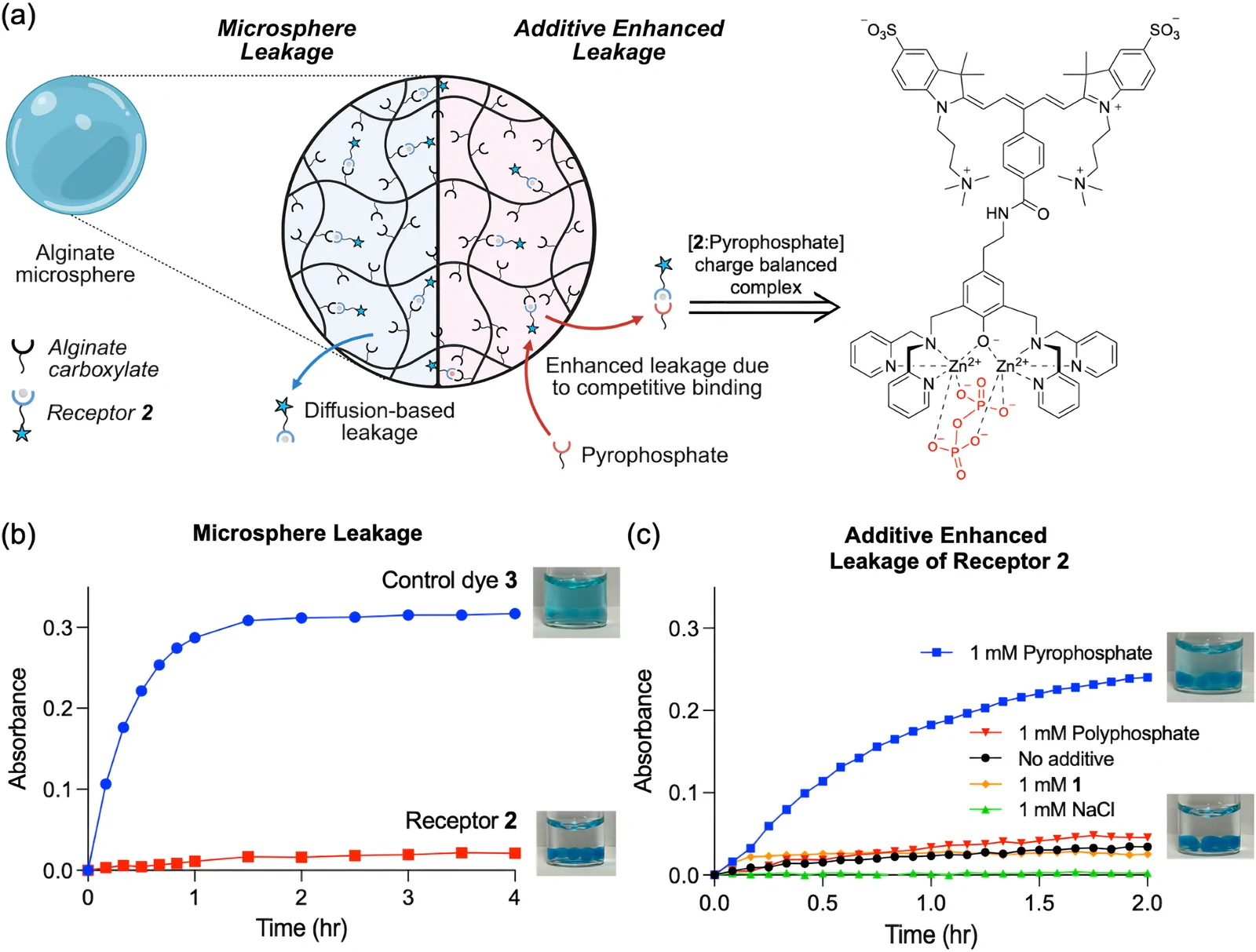
(a) Schematic picture showing retention of receptor 2 within a Ca-crosslinked alginate microsphere due to association with alginate carboxylate groups. Addition of pyrophosphate displaces 2 from the alginate carboxylates and forms a charge-balanced [2:pyrophosphate] complex with enhanced leakage from the microsphere. (b) Leakage of receptor 2 or control dye 3 from preloaded microspheres into the external solution, as indicated by the increase in 643 nm absorption for the exterior solution. (c) Leakage of receptor 2 from separate batches of preloaded microspheres that were incubated in the presence of different additives (1 mM). All experiments in HEPES buffer (10 mM, pH 7.2).

The next step in the project was to measure leakage of molecular payload from Ca-crosslinked alginate hydrogel microspheres.^42^ The previous literature on leakage of small molecular payload from alginate microspheres has established that anionic or uncharged hydrophilic molecules escape with little apparent interaction with the alginate strands,^7,43^ whereas cationic dyes,^44–46^ or cationic drugs,^47–50^ leak more slowly due to their moderate affinity. We hypothesized that ZnBDPA receptors would be strongly retained within Ca-crosslinked alginate hydrogels due to binding with the alginate carboxylate anions, as shown in Scheme 1a and Fig. 3a. This hypothesis was tested by preparing a set of homologous Ca-crosslinked alginate hydrogel microspheres (2.3 mm average diameter) that were preloaded with either ZnBDPA receptor 2 or the control dye 3. Both compounds absorb strongly at 643 nm and the first set of leakage experiments placed ten identical preloaded microspheres in an external solution of HEPES buffer (3 mL, 10 mM, pH 7.2) and monitored the increase in 643 nm absorption for the external solution. As shown in Fig. 3b, leakage of control dye 3 from the preloaded microspheres into the external solution was extensive and complete after two hours. In contrast, leakage of ZnBDPA receptor 2 was <5% over the same time. The photographs in Fig. 3b provide clear visual evidence of the large difference in leakage from the microspheres after four hours; there is complete leakage of control dye 3 from the preloaded microspheres, whereas receptor 2 is strongly retained within the microspheres.

Next, we conducted experiments that tested the capacity of molecular additives to enhance the leakage of ZnBDPA receptor 2. Shown in Fig. 3c are the leakage profiles for separate samples of alginate microspheres that were each preloaded with receptor 2 and then allowed to incubate in an external solution containing one of the following solution additives (1 mM), pyrophosphate, NaCl, ZnBDPA receptor 1, or polyphosphate. Pyrophosphate was the only external solution additive that greatly enhanced leakage of 2 from the preloaded microspheres. Indeed, the pyrophosphate-induced leakage of 2 continued over time until completion after 24 hours, indicating full displacement of 2 from the microspheres (Fig. S6) with very little visible change in microsphere morphology. We attribute this pyrophosphate-induced leakage effect to the combination of two factors: (a) pyrophosphate has higher affinity than alginate for receptor 2,† and (b) pyrophosphate is small enough to diffuse into a microsphere and displace 2 from the alginate strands and form a charge-balanced [2:pyrophosphate] complex that readily diffuses out of the microsphere (Fig. 3a).

To the best of our knowledge this is the first example of affinity-based chemical displacement of molecular payload from a preloaded Ca-crosslinked alginate hydrogel. In contrast to the microsphere leakage induced by added pyrophosphate, addition of polyphosphate to the external solution did not have any effect on microsphere leakage of receptor 2 (Fig. 3c), which is a striking difference because polyphosphate has moderately high affinity for receptor 2 (see Fig. 1). It appears that the large hydrodynamic size and polyanionic charge of polyphosphate molecules prevent them from accessing the bound receptor 2 within the Ca-crosslinked alginate hydrogel and forming a suitable complex with 2 that can diffuse out the microspheres. Limited penetration of long anionic polymers into the core of alginate microspheres has been previously documented,^11^ and systematic studies have shown that molecules with molecular weight >2 × 10^4^ do not diffuse freely in and out of a Ca-crosslinked alginate hydrogel.^51^

Mechanistically, the inability of polyphosphate to trigger leakage of 2 from the microspheres is evidence that the added pyrophosphate does not induce leakage of 2 by simply chelating the Ca^2+^ cations and destroying the cross-linked microsphere structure. If this microsphere degradation mechanism was significant then the added polyphosphate would likely produce the same leakage as added pyrophosphate since they have similar affinity for Ca^2+^. Visual inspection of the microspheres at the end of all the leakage experiments revealed no discernible morphology change, indicating no major disruption of the Ca-crosslinked structure. Moreover, an analysis of the external solutions found that the presence of pyrophosphate or polyphosphate did not promote Ca^2+^ leaching from Ca-crosslinked microspheres (Fig. S7). A final noteworthy result in Fig. 3b is the inability of added ZnBDPA receptor 1 (1 mM) to displace trapped receptor 2 from the microspheres. This suggests that not all the internally located alginate carboxylates are bound by the trapped receptor 2 and a moderate number of alginate carboxylates remain available for occupancy by added receptor 1 without the need to displace receptor 2.

### Loading of fluorescent ZnBDPA receptor 2 into empty alginate hydrogel microspheres

**Fig. 4:**
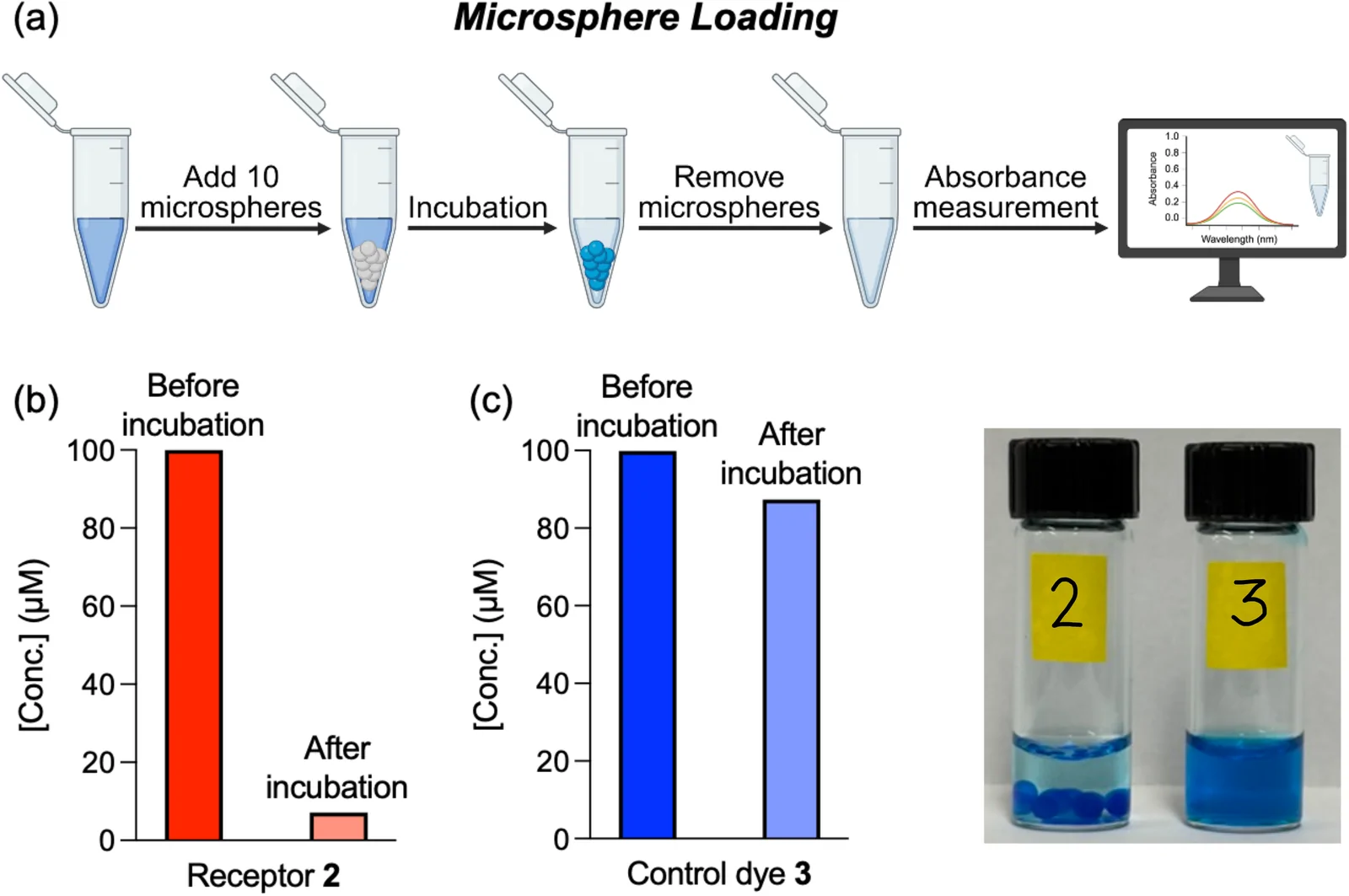
(a) Ca-crosslinked alginate microsphere loading study using absorption spectroscopy to measure the external solution concentration before and after incubation with ten “empty” Ca-crosslinked microspheres. External solution concentration data for (b) receptor 2, and (c) control dye 3 (each started with 100 µM solutions). Each experiment was conducted three times, the error bars indicating ±SEM are too small to visualize. The photograph shows that after the 24-hour incubation there was substantially more loading of blue-colored receptor 2 into the empty microspheres than control dye 3. In each case, the external solution was HEPES buffer (10 mM, pH 7.2).

Ca-crosslinked alginate microspheres were prepared without any trapped additive and subsequently allowed to incubate in either an external solution containing fluorescent receptor 2 or control dye 3. Loading into these “empty” microspheres was measured by homologous experiments that employed either absorption spectroscopy or fluorescence imaging. The absorption spectroscopy experiments incubated a set of ten identical microspheres in separate external buffer solutions containing 100 µM of receptor 2 or control dye 3. Loading into the microspheres was indicated by the decreased dye absorption in the external solution (Fig. 4a). The bar graphs in Fig. 4b and c reflect a substantial difference in microsphere loading efficiencies, with the ten microspheres combining to take up >95% of 2 but <10% of 3. The photograph in Fig. 4c clearly shows much greater transfer of blue-colored 2 from the external buffer solution into the empty Ca-crosslinked alginate microspheres.

**Fig. 5:**
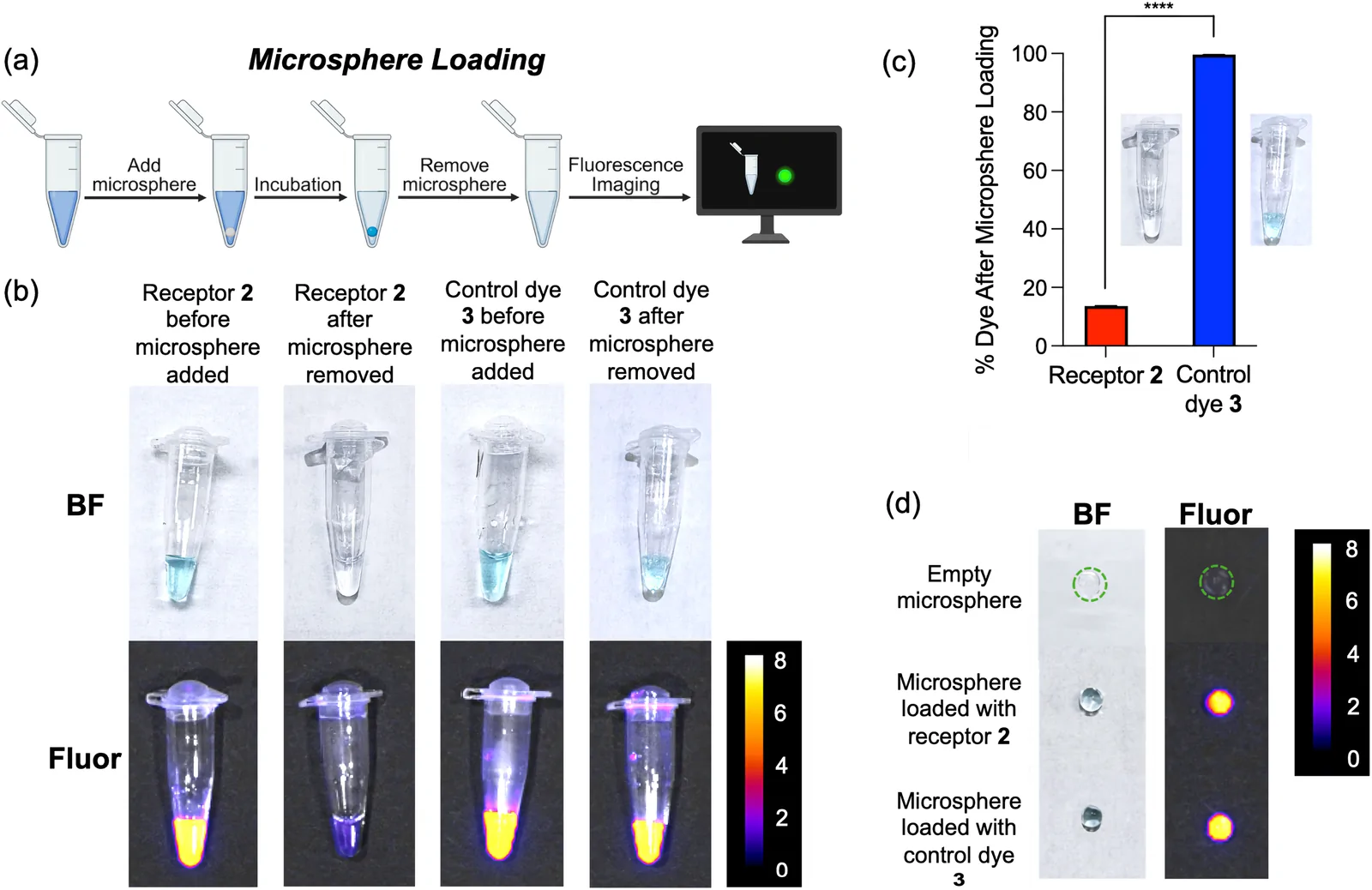
(a) Schematic of microsphere loading study monitored by fluorescence imaging. An “empty” Ca-crosslinked alginate microsphere (2 mm in diameter) was submerged in an external solution containing either 2 or 3 (10 µM in HEPES 10 mM, pH 7.2) and removed from the solution at various time points for imaging. (b) Representative brightfield and fluorescence images of Eppendorf tubes containing external solutions of 2 or 3 before adding the microsphere and 24 hours after incubation and microsphere removal. (c) Quantitative analysis of the percent dye remaining in the external solution after microsphere incubation and removal. Each experiment was conducted three times, error bars are too small to visualize, ****p < 0.0001. (d) Representative brightfield and fluorescence images of an empty microsphere, and two separated microspheres after 24 hour incubation in a solution of 2 or 3 (10 µM in HEPES 10 M, pH 7.2), respectively. Fluorescence images acquired using ex: 605 nm, em: 670 nm, with fluorescence intensity scale in arbitrary units.

A separate set of fluorescence imaging experiments incubated a single empty microsphere in an external buffer solution containing 10 µM of receptor 2 or control dye 3 and determined, (a) the decrease in dye fluorescence intensity at 666 nm in the external solution and (b) the appearance of dye fluorescence within the microsphere (Fig. 5a). The Eppendorf tube images in Fig. 5b and associated quantification in Fig. 5c show that after 24 hours there was almost ∼85% removal of receptor 2 from the external solution, but negligible removal of control dye 3. Images taken at much shorter incubation timepoints showed that transfer of receptor 2 from the external solution into the microsphere was complete within one hour (Fig. S8). The deep-red fluorescence images of the two separated microspheres in Fig. 5d are false-colored to indicate the mean pixel intensity (MPI) for each microsphere. Interestingly, the MPI for each microsphere was very similar even though there was about seven times more fluorophore in the microsphere containing receptor 2 compared to the microsphere containing control dye 3. This fluorescence imaging effect was confirmed with an independent set of microsphere loading experiments that used a high throughput, multiwell array fluorescence imaging protocol (Fig. S9). The lower-than-expected fluorescence intensity for the microspheres containing a high level of receptor 2 is attributed to a combination of fluorescence self-quenching and inner filter effect caused by close packing of high levels of 2 within the microsphere.

The multiwell array fluorescence imaging protocol was used to determine how much receptor 2 loading was decreased when the external solution contained inorganic and biomolecular anions that compete for the binding of receptor 2. In Fig. S10 are the results for external solutions composed of HEPES buffer, PBS, saline, 10% serum, and 100% serum. As expected, changing the external solution from HEPES to a more competitive solution led to reduced transfer of 2 into the microsphere. Nonetheless, fluorescence emission from the loaded microsphere was still high because the lower amount of 2 in the microsphere produced less fluorescence self-quenching and inner filter effect. Finally, it is worth emphasizing that visual inspection of all microspheres after a 24-hour incubation period showed no evidence of altered microsphere diameter or morphology.

### Targeting Ca-crosslinked alginate microsphere implanted in a living mouse

The last step in the project was to determine if receptor 2 could selectively target and accumulate in a Ca-crosslinked alginate microsphere that was implanted in a living mouse. Before commencing the mouse experiments, a set of cell metabolic activity measurements were conducted to prove that receptor 2 or control dye 3 were not toxic to cultured cells (human pulmonary fibroblasts or hepatocellular carcinoma cells) (Fig. S11).

**Fig. 6:**
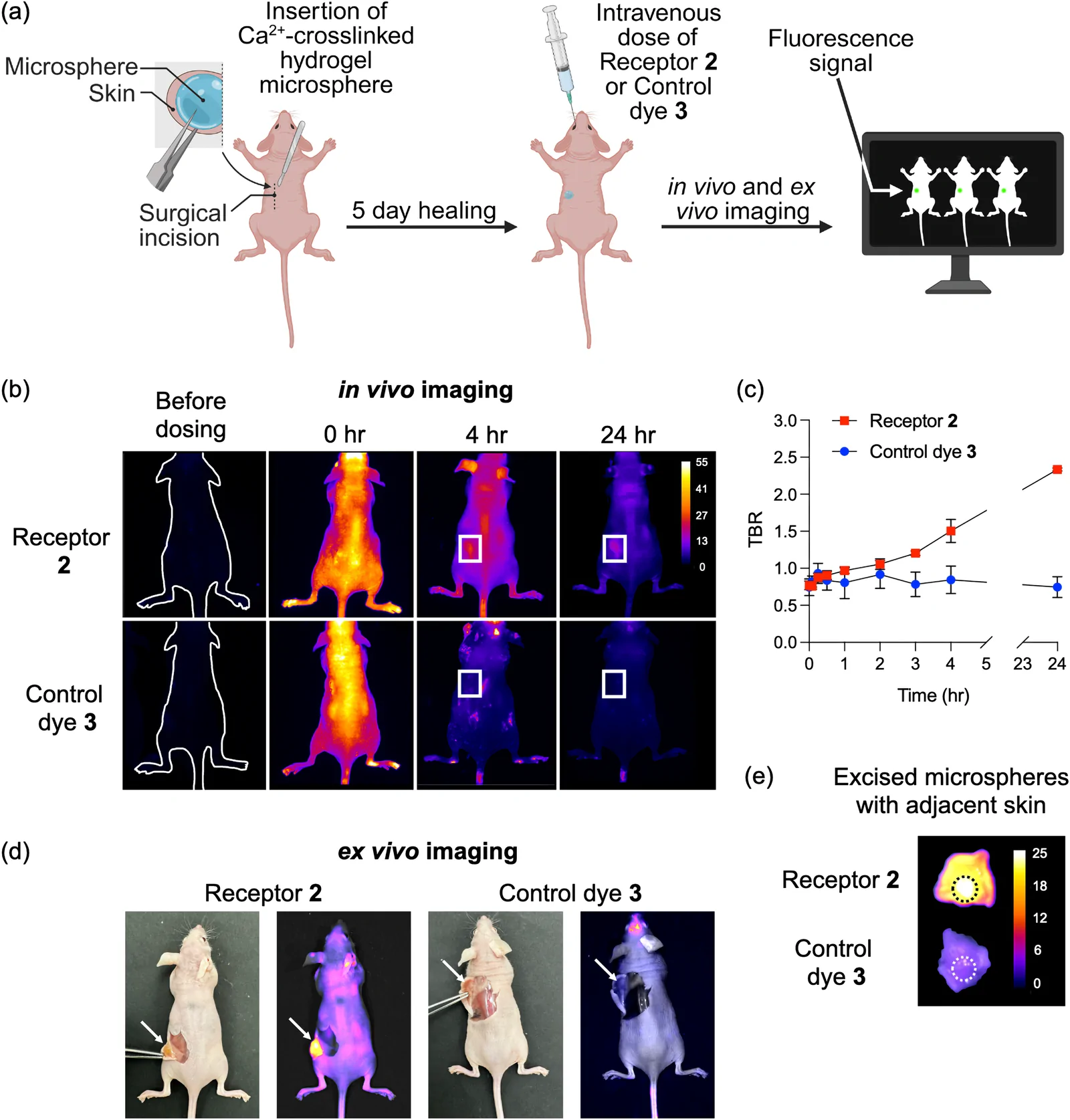
(a) Experimental workflow for implantation of a Ca-crosslinked alginate microsphere and subsequent mouse fluorescence imaging. (b) Representative fluorescence images of two separate nude mice, each implanted with a Ca-crosslinked alginate microsphere and imaged at different time points after intravenous dosing with receptor 2 or control dye 3 (100 nmol per mouse); the fluorescence intensity scale bar applies to all mouse images. (c) Plot of average Target-Background-Ratio (TBR) for living mice over time after dosing; *N* = 4 for 2, *N* = 3 for 3, with error bars indicating ± SEM. (d) Representative *ex vivo* images of mice sacrificed at 24 hours after dosing with 2 or 3; the white arrow shows the exposed microsphere at the excised implantation site. (e) Representative *ex vivo* fluorescence images of excised microsphere (circled) with adjacent skin from mice dosed with receptor 2 or control dye 3; the fluorescence intensity scale bar applies to both images. All fluorescence images were acquired using an *in vivo* imaging station (ex: 605 nm, em: 670 nm).

The mouse experiments followed the workflow in Fig. 6a that was approved by the Institutional Animal Care and Use Committee. In short, a set of identical Ca-crosslinked alginate microspheres (4.0 mm diameter) were prepared without any trapped additive, and each microsphere was implanted subcutaneously in the left dorsal area of a cohort of female athymic nude mice. After a five-day recovery period (Fig. S12), each mouse was given a single intravenous dose (50 µL, 100 nmol) of receptor 2 or control dye 3. Whole body fluorescence images of the living mice were acquired at eight incremental post-injection time points up to 4 hours and again at 24 hours (Fig. 6b and S13–S14) and the digital whole-body images were analyzed to give a plot of *in vivo* Target-Background-Ratio (TBR) over time (Fig. 6c). The mice were sacrificed at 24 hours, imaged post-sacrifice (Fig. 6d and e), and the final biodistribution determined by fluorescence imaging of the excised organs and tissues (Fig. S15).

Examination of the *in vivo* mouse fluorescence images reveals a clear difference; the living mice dosed with receptor 2 exhibited a time dependent increase in TBR at the site of the implanted microsphere, reaching a value of 2.5 after 24 hours, whereas there was no significant change in TBR for the mice dosed with control dye 3 (Fig. 6c). Rapid renal clearance of control dye 3 from the mice was quite apparent from the blue color of the mouse urine very soon after dosing. In contrast, the residence time of receptor 2 in the mouse circulation was noticeably longer as reflected by the difference in whole-body mouse fluorescence images at 4 hours after dosing (Fig. 6b). *Ex vivo* fluorescence imaging of the excised organs, tissue, and microsphere confirmed the much higher accumulation of receptor 2 in the implanted microsphere compared to 3 (Fig. 6e and S15). Visual inspection of each excised microsphere revealed partial erosion of the spherical structure and some physical integration of the hydrogel material with the surrounding connective tissue. This change in microsphere morphology after seven days of implantation is consistent with previous reports that the mechanical structures of implanted Ca-crosslinked alginate microspheres are partly disrupted after a week or so in a living subject.^52^ Localized diffusion of polyanionic alginate strands from the partially eroded microsphere into the nearby connective tissue explains why there is also slightly more receptor 2 in the mouse skin and tissue surrounding the implanted microsphere compared to skin that is distal to the implanted microsphere (Fig. S15). It is possible that a small fraction of receptor 2 binds to anionic cells or anionic biopolymers, such as GAGs, that are attracted to the implanted microsphere as part of a foreign body response (FBR). This scenario is unlikely to be the major reason for accumulation of dosed receptor 2 in and around the implanted 4 mm diameter microsphere because pure unmodified alginate is highly biocompatible and it is known that spherical alginate implants with diameters above 1.5 mm do not elicit a strong FBR, especially in immunodeficient mice like those used in this one-week study.^53^ Moreover, histological and fluorescence imaging of the excised microsphere and surrounding tissue confirm the absence of immune cells and the localization of receptor 2 to the implanted alginate (Fig. S16).

Regarding the biocompatibility of the dyes used in this study, it is important to emphasize that the structure of control dye 3 (which is also the fluorophore component within receptor 2) has “zwitterionic” character.^54^ That is, its spatial distribution of opposing cationic and anionic residues favors low affinity for biological surfaces such as cell membranes and blood proteins. This designed “non-stick” property was confirmed with titration experiments showing that 2 and 3 have weak affinity for albumin protein (Fig. S18) and explains why control dye 3 exhibits rapid excretion from the mouse and low retention in the implanted microsphere. It also explains why mice treated with receptor 2 or control dye 3 show no histological evidence of tissue damage in the liver and kidney clearance organs.‡‡Thin histology sections of liver and kidney from mice treated with receptor 2 or control dye 3 were stained with haematoxylin and eosin (H&E) and imaged. As shown by the representative micrographs in Fig. S17 there were no morphological abnormalities or signs of tissue damage, which is consistent with the high biocompatibility of unmodified alginate and the low cell toxicity of 2 and 3 (Fig. S11).

The impressive *in vivo* targeting performance by receptor 2 is consistent with a large body of published work over the last two decades demonstrating that fluorescent ZnBDPA probes are effective injectable agents for imaging negatively charged biological surfaces within sites of disease in a living subject.^23^ This literature also includes reports that ZnBDPA conjugates with an appended antibiotic or anticancer drug can locate their pharmaceutical targets in complex biological media.^55,56^ Thus, ZnBDPA conjugates can likely be used to deliver a wide range of molecular payload to an implanted alginate hydrogel. Chemotherapy applications can be envisioned where an alginate hydrogel is implanted at a site of disease and subsequently used as a depot for repeated loading and controlled release of ZnBDPA-drug conjugates. Alternatively, an implanted alginate hydrogel could encapsulate a cluster of living cells for tissue engineering, and a subsequent dosing protocol could utilize ZnBDPA-conjugates to deliver nutrients for facilitated cell growth or cell signalling. Beyond health and medicine, there are possible applications in cell-based manufacturing and energy production where ZnBDPA-conjugates could be used to selectively deliver nutrients to alginate-encapsulated cells within complex multicell cultures.^57,58^

This prototype study employed structurally simple Ca-crosslinked alginate hydrogel microspheres, which are easy to fabricate and very useful for proof-of-concept studies, but they only have moderate *in vivo* stability.^52^ Future studies can pursue alginate hydrogel stabilization and surface coating strategies that are known to extend *in vivo* lifetimes to several months with negligible FBR.^59,60^ This will lead to long-lasting alginate implants that can be deployed as targetable depots for repeated loading and controlled release of ZnBDPA conjugates as molecular payload. In addition, the ZnBDPA structure can be tailored for optimal pharmacokinetics and alginate targeting performance. Most likely, the concept of multivalency can be exploited to increase the affinity of a ZnBDPA conjugate for an alginate hydrogel. Particularly alluring is the possibility of ultrahigh affinity conjugates with multiple ZnBDPA units.^23^ Finally, we note that this general supramolecular targeting strategy may be applicable to other common classes of carboxylate-containing anionic polysaccharides, such as pectin or hyaluronate,^61,62^ that are used often to form implantable hydrogels or mixed hydrogel composites.^63^

## Conclusions

This study establishes a novel supramolecular methodology for delivering molecular payload to Ca-crosslinked alginate hydrogel microspheres. We conceptualized the alginate polymer scaffold as an anionic supramolecular target and designed the fluorescent ZnBDPA receptor 2 to bind the carboxylate groups along the alginate strands. Solution-state and microsphere assays confirmed that added receptor 2 accumulates within the hydrogel network and is strongly retained, unlike the unconjugated control dye 3 that rapidly diffuses into the surrounding solution. Furthermore, the bound receptor 2 can be displaced from a microsphere by introducing pyrophosphate as a competitive binder. *In vivo* fluorescence imaging further validated this approach by demonstrating that intravenously administered receptor 2 selectively targets a subcutaneously implanted alginate microsphere in a living mouse. These findings present a promising path towards implanted alginate hydrogels as targetable depots for various types of medical, diagnostic, or tissue engineering applications.^2^

## Ethical statement

The animal experimental protocol for this study was approved by the Institutional Animal Care and Use Committee (IACUC) of the University of Notre Dame (Approval No. 25-09-9572). All experimental procedures were conducted in strict accordance with national regulations and international guidelines for animal research.

## Author contributions

Conceptualization: H. C., R. G., B. S.; investigation, methodology, visualization, and validation: H. C., R. G., J. C., C. P., C. D.; writing – original draft, review, and editing: H. C., R. G, B. S.; supervision and funding acquisition: B. S., D. H.-P.

## Conflicts of interest

There are no conflicts to declare.

## Supplementary Material

SC-OLF-D6SC05110C-s001

## Data Availability

The data supporting this article have been included as part of the supplementary information (SI). Supplementary information: characterization, experimental procedures, microsphere loading and leakage data, mouse imaging data. See DOI: https://doi.org/10.1039/d6sc05110c.

## References

[cit1] Lee K., Mooney D. J. (2012). Prog. Polym. Sci..

[cit2] Dong S., An S., Saiding Q., Chen Q., Liu B., Kong N., Chen W., Tao W. (2025). Chem. Rev..

[cit3] Ching S. H., Bansal N., Bhandari B. (2017). Crit. Rev. Food Sci. Nutr..

[cit4] Cao L., Lu W., Mata A., Nishinari K., Fang Y. (2020). Carbohydr. Polym..

[cit5] Ghanta R. K., Aghlara-Fotovat S., Pugazenthi A., Ryan C. T., Singh V. P., Mathison M., Jarvis M. I., Mukherjee S., Hernandez A., Veiseh O. (2020). Biomater. Sci..

[cit6] Ramezani M., Baheiraei N., Bathaie S. Z., Razavi M., Naderi N. (2025). Int. J. Biol..

[cit7] Fleten K. G., Hyldbakk A., Einen C., Benjakul S., Strand B. L., Davies C. de L., Mørch Ý., Flatmark K. (2022). Mar. Drugs.

[cit8] Palvai S., Moody C. T., Pandit S., Brudno Y. (2021). Mol. Pharm..

[cit9] Czuban M., Srinivasan S., Yee N. A., Agustin E., Koliszak A., Miller E., Khan I., Quinones I., Noory H., Motola C. (2018). et al.. ACS Cent. Sci..

[cit10] Abune L., Wang Y. (2021). Trends Pharmacol. Sci..

[cit11] Brudno Y., Silva E. A., Kearney C. J., Lewin S. A., Miller A., Martinick K. D., Aizenberg M., Mooney D. J. (2014). Proc. Natl. Acad. Sci. U. S. A..

[cit12] Omtvedt L. A., Kristiansen K. A., Strand W. I., Aachmann F. L., Strand B. L., Zaytseva-Zotova D. S. (2021). J. Biomed. Mater. Res., Part A.

[cit13] Wu J. P. J., Cheng B., Roffler S. R., Lundy D. J., Yen C. Y. T., Chen P., Lai J. J., Pun S. H., Stayton P. S., Hsieh P. C. H. (2016). Sci. Transl. Med..

[cit14] Abune L., Davis B., Wang Y. (2021). Wiley Interdiscip. Rev.:Nanomed. Nanobiotechnol..

[cit15] Addonizio C. J., Gates B. D., Webber M. J. (2021). Bioconjug. Chem..

[cit16] Rivera-Delgado E., Nam J. K., Von Recum H. A. (2017). ACS Biomater. Sci. Eng..

[cit17] Chen J., Li S., Zhang Y., Wang W., Zhang X., Zhao Y., Wang Y., Bi H. (2017). Adv. Healthcare Mater..

[cit18] Masoumi Shahrbabak S., Jalali S. M., Fathabadi M. F., Tayebi-Khorrami V., Amirinejad M., Forootan S., Saberifar M., Fadaei M. R., Najafi Z., Askari V. R. (2025). Int. J. Pharm.:X.

[cit19] Mei X., Chang Y., Shen J., Zhang Y., Xue C. (2020). Carbohydr. Polym..

[cit20] Mei X., Tao W., Sun H., Liu H., Chen G., Zhang Y., Xue C., Chang Y. (2024). Int. J. Biol. Macromol..

[cit21] Ji S., Tian X., Li X., She Q. (2023). J. Biol. Chem..

[cit22] Parvez A., Baum D. A. (2024). ACS Biomater. Sci. Eng..

[cit23] Rice D. R., Clear K. J., Smith B. D. (2016). Chem. Commun..

[cit24] Uchinomiya S., Ojida A., Hamachi I. (2014). Inorg. Chem..

[cit25] Ngo H. T., Liu X., Jolliffe K. A. (2012). Chem. Soc. Rev..

[cit26] Kumar N., Roopa, Bhalla V., Kumar M. (2021). Coord. Chem. Rev..

[cit27] Ojida A., Honda K., Shinmi D., Kiyonaka S., Mori Y., Hamachi I. (2006). J. Am. Chem. Soc..

[cit28] Ojida A., Fujishima S., Honda K., Nonaka H., Uchinomiya S., Hamachi I. (2010). Chem. – Asian J..

[cit29] O'Neil E. J., Jiang H., Smith B. D. (2013). Supramol. Chem..

[cit30] Morsby J. J., Zhang Z., Burchett A., Datta M., Smith B. D. (2024). Chem. Sci..

[cit31] Xiao S., Turkyilmaz S., Smith B. D. (2013). Tetrahedron Lett..

[cit32] Su Han M., Kim D. H. (2002). Angew Chem. Int. Ed. Engl..

[cit33] Hanshaw R. G., O'Neil E. J., Foley M., Carpenter R. T., Smith B. D. (2005). J. Mater. Chem..

[cit34] Manabe Y., Tsutsui M., Hirao K., Kobayashi R., Inaba H., Matsuura K., Yoshidome D., Kabayama K., Fukase K. (2022). Chem. – Eur. J..

[cit35] Hu X., Wang Q., Liu Y., Liu H., Qin C., Cheng K., Robinson W., Gray B. D., Pak K. Y., Yu A. (2014). et al.. Biomaterials.

[cit36] He H., Huang C., Huang H., Lan N., Liu S., Luo Y., Zheng L., Liu G., Qin Z., Zhao J. (2025). Biomaterials.

[cit37] Honda K., Fujishima S. H., Ojida A., Hamachi I. (2007). ChemBioChem.

[cit38] Chiaramonte J., Cheney H. B. D., Smith B. D. (2025). Supramol. Chem..

[cit39] Mamchits E. G., Nasimova I. R., Makhaeva E. E., Khokhlov A. R. (2006). Polym. Sci., Ser. A.

[cit40] Lipatova I. M., Yusova A. A., Lukyanets E. A. (2018). J. Photochem. Photobiol..

[cit41] Khouri S. J., Buss V. (2011). Biophys. J..

[cit42] Dhamecha D., Movsas R., Sano U., Menon J. U. (2019). Int. J. Pharm..

[cit43] Dolmat M., Thomas C., Kozlocskaya V., Kharlampieva E. (2022). J. Chem. Educ..

[cit44] Solovieva A., Kopylov A., Cherkasova A., Shershnev I., Kaplin V., Timofeeva V., Akovantseva A., Savko M., Gulin A., Zarkhina T. (2024). et al.. Polymers.

[cit45] Dominguez M. A., Etcheverry M., Zanini G. P. (2019). Adsorption.

[cit46] Asadi S., Eris S., Azizian S. (2018). ACS Omega.

[cit47] Saadatidizaji Z., Sohrabi N., Mohammadi R., Amini-Fazl M. S. (2024). Int. J. Biol. Macromol..

[cit48] Luu C. H., Nguyen G., Le T. T., Nguyen T. M. N., Giang Phan V. H., Murugesan M., Mathiyalagan R., Jing L., Janarthanan G., Yang D. C. (2022). et al.. Gels.

[cit49] Shee M., Lal Banerjee S., Dey A., Das Jana I., Basak P., Mandal M., Mondal A., Kumar Das A., Das C. N. (2024). Int. J. Pharm..

[cit50] Bouhadir K. H., Alsberg E., Mooney D. J. (2001). Biomaterials.

[cit51] Tanaka H., Matsumura M., Veliky I. A. (1984). Biotechnol. Bioeng..

[cit52] Nunamaker E. A., Purcell E. K., Kipke D. R. (2007). J. Biomed. Mater. Res., Part A.

[cit53] Veiseh O., Doloff J. C., Ma M., Vegas A. J., Tam H. H., Bader A. R., Li J., Langan E., Wyckoff J., Loo W. S. (2015). et al.. Nat. Mater..

[cit54] Choi H. S., Gibbs S. L., Lee J. H., Kim S. H., Ashitate Y., Liu F., Hyun H., Park G., Xie Y., Bae S. (2013). et al.. Nat. Biotechnol..

[cit55] Lo C. F., Chiu T. Y., Liu Y. T., Pan P. Y., Liu K. L., Hsu C. Y., Fang M. Y., Huang Y. C., Yeh T. K., Hsu T. A. (2022). et al.. J. Med. Chem..

[cit56] Sarkar P., Xu W., Vázquez-Hernández M., Dhanda G., Tripathi S., Basak D., Xie H., Schipp L., Dietze P., Bandow J. E. (2024). et al.. Chem. Sci..

[cit57] Soares R. C., Zangirolami T. C., Giordano R. L. C., Demeke M. M., Thevelein J. M., Milessi T. S. (2022). Polymers.

[cit58] Levä T., Rissanen V., Nikkanen L., Siitonen V., Heilala M., Phiri J., Maloney T. C., Kosourov S., Allahverdiyeva Y., Mäkelä M. (2023). Biomacromolecules.

[cit59] Liu Q., Chiu A., Wang L. H., An D., Zhong M., Smink A. M., de Haan B. J., de Vos P., Keane K., Vegge A. (2019). et al.. Nat. Commun..

[cit60] Kavand A., Noverraz F., Gerber-Lemaire S. (2024). Pharmaceutics.

[cit61] Cao L., Lu W., Mata A., Nishinari K., Fang Y. (2020). Carbohydr. Polym..

[cit62] Park H., Woo E. K., Lee K. Y. (2014). J. Contr. Release.

[cit63] Kass L. E., Nguyen J. (2022). Wiley Interdiscip. Rev.:Nanomed. Nanobiotechnol..

[cit64] Svane S., Kjeldsen F., McKee V., McKenzie C. J. (2015). Dalton Trans..

[cit65] Zhu Z., Zhou J., Li Z., Yang C. (2015). Sens. Actuators, B.

