# Supplementary material for: Targeting calcium-crosslinked alginate hydrogel in a living subject using a synthetic anion receptor
Source: Chem Sci. 2026 Aug 25;17(40):19376–86. doi: 10.1039/d6sc05110c (PMC13524122; doi:10.1039/d6sc05110c)
Supplement: Supplementary file 1 [file SC-017-D6SC05110C-s001.pdf]

Supplementary Information (SI) for Chemical Science.
This journal is © The Royal Society of Chemistry 2026

Electronic Supplementary Information for:

Targeting calcium-crosslinked alginate microspheres in a living subject using a synthetic anion receptor

Hunter B. D. Cheney, [a] Rananjaya S. Gamage, [a, b] Jon Chiaramonte, [a] Catherine G. Price, [a] Colin Davidson, [a] Donny Hanjaya-Putra, [b] and Bradley D. Smith [a] *

[a] Department of Chemistry and Biochemistry, University of Notre Dame, Notre Dame, IN USA 46556
[b] Bioengineering Program, Department of Aerospace and Mechanical Engineering, University of Notre Dame, Notre Dame, IN USA 46556
*email: smith.115@nd.edu

Table of contents

1. General Methods and Materials S2
2. Synthesis S2
3. Spectral Data for Receptor **2** and Control Dye **3** S8
4. Pyrocatechol Violet Displacement Studies S9
5. Alginate Microsphere Studies S16
6. Cell Metabolic Activity Measurements S21
7. Mouse Microsphere Implant Studies S22
8. Albumin Binding Measurements S28
9. References S29

1. General Methods and Materials

All reagents and solvents were purchased from Fisher Scientific, Alfa Aesar, ThermoScientific, Oakwood Chemical, Sigma, or Fluka Analytical, and used without further purification unless stated otherwise. Alginic acid sodium salt (70% guluronic acid and 30% mannuronic acid) was purchased from Sigma-Aldrich (Cat. No: 71238). Alginic acid sodium salt medium viscosity (35% guluronic acid and 65% mannuronic acid) was also purchased from Sigma-Aldrich (Cat. No.: A2033). Sodium polyphosphate was purchased from ThermoScientific (Cat. No: 390932500). Anion stock solutions (10 mM unless otherwise specified) were prepared as sodium salts in HEPES buffer (10 mM, pH 7.2) and stored at room temperature. Stock solutions of compounds **1 - 3** and pyrocatechol violet were each prepared at 1 mM in HEPES buffer (10 mM, pH 7.2) and stored at -20ºC. UV-Vis absorption spectra were recorded using an Evolution 201 spectrophotometer (Thermo Fisher Scientific, WA, USA). Fluorescence spectra were collected using a Fluoromax Plus C fluorometer (Horiba Instruments, Kyoto, Japan).

2. Synthesis

Apo-Receptor **1** is a known compound and was prepared according to **Scheme S1**.[1]

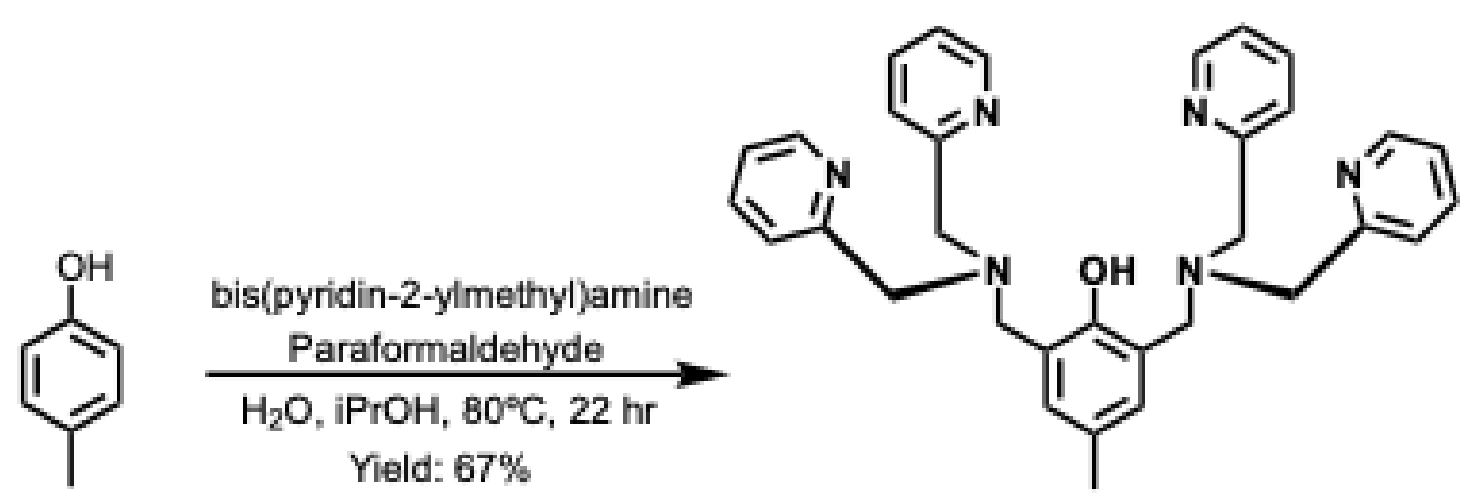

Scheme S1. Synthesis of apo-receptor **1**.

2,2'-Dipicolylamine (2.810 g, 14.1 mmol, 2.5 eq) and paraformaldehyde (0.677 g, 22.5 mmol, 4 eq) were added to a mixture of 15 mL isopropanol and 25 mL of water. The mixture was heated to 80 °C for 30 minutes. After this, the reaction was allowed to cool for a bit and then, p-cresol (0.608 g, 5.62 mmol, 1 eq) was added and the mixture was heated to 80 °C for 22 hours. The reaction mixture was allowed to cool to room temperature. The solvents were then evaporated using a rotary evaporator. The residue was then dissolved in ethyl acetate and washed with a saturated solution of sodium bicarbonate, followed by washing with water and then brine. The ethyl acetate solution was then dried with anhydrous sodium sulfate and filtered. The ethyl acetate was then evaporated, and the residue was purified by column chromatography using 0-2% methanol in chloroform to give apo-receptor **1** as a pale-yellow solid (1.989 g, 3.75 mmol, 67% yield). Apo-receptor **1** was converted into receptor **1** by mixing with 2.2 molar equivalents of $Zn(NO_3)_2$ in methanol and stirring at room temperature for 30 min. The solvent was evaporated to obtain receptor **1**.

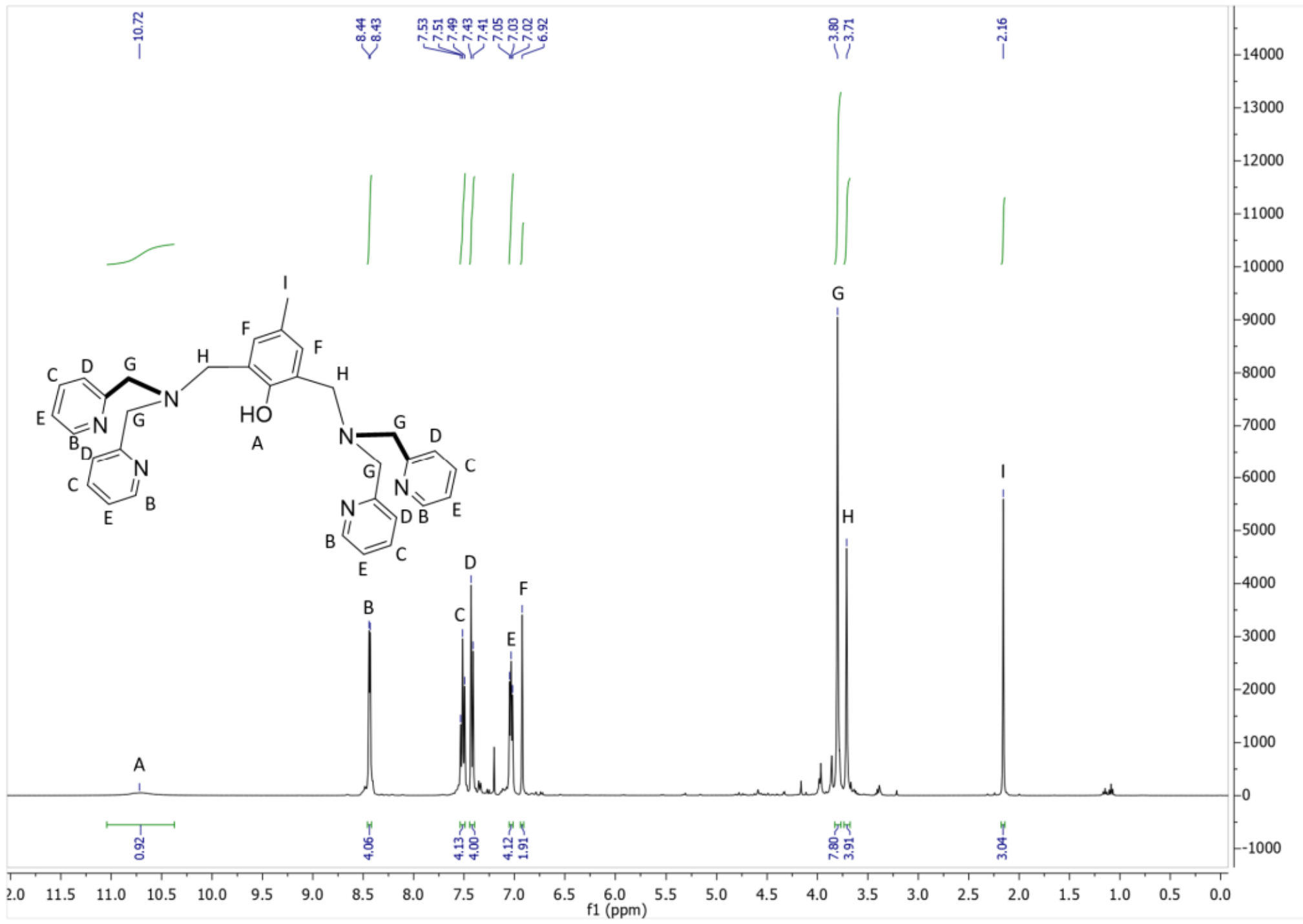

^{1}H NMR spectrum (400 MHz, $CDCl_3$, 25°C) of apo-receptor **1**

Control dye **3** is a known compound and was prepared according to the sequence in **Schemes S2-S4**.[2,3]

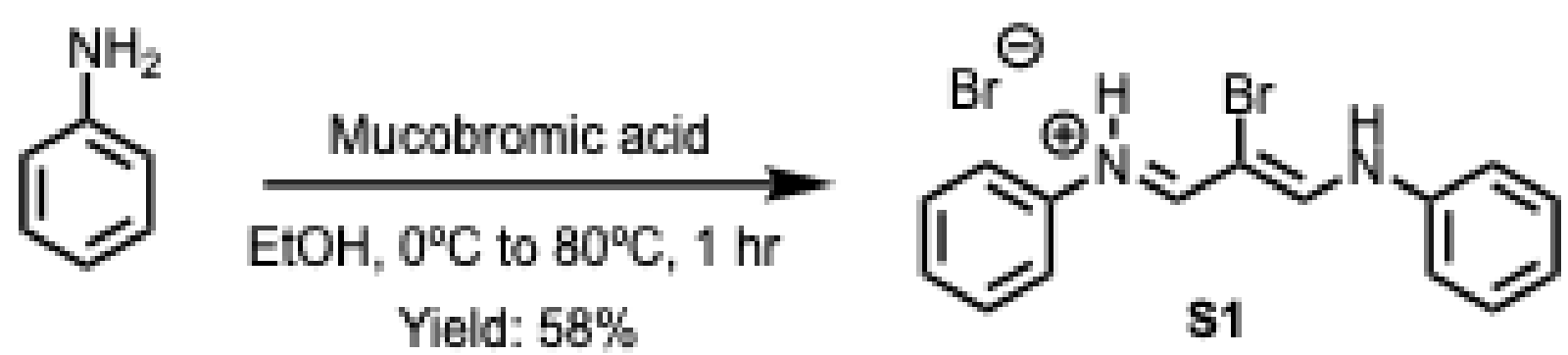

Scheme S2. Synthesis of **S1**.

S1: Prepared by a known method.[2,3] Aniline (3.45 mL) was dissolved in 15 mL of ethanol (95%) and placed in an ice bath. Separately, 4.9955 g of mucobromic acid was dissolved in 15 mL of ethanol (95%). The mucobromic acid solution was then added slowly dropwise to the aniline solution at 0 °C. After the addition, the mixture was allowed to warm to room temperature and stir for 1 hour. The mixture was then heated to evaporate approximately half of the ethanol present. The mixture was cooled in ice and then filtered to give **S1** (4.2397 g, 58.4%). ^{1}H NMR (400 MHz, $CDCl_3$, 25 °C) δ 11.49 (s, 2H), 9.21 (s, 2H), 7.61 (d, 4H), 7.52 (t, 4H), 7.35 (t, 2H).

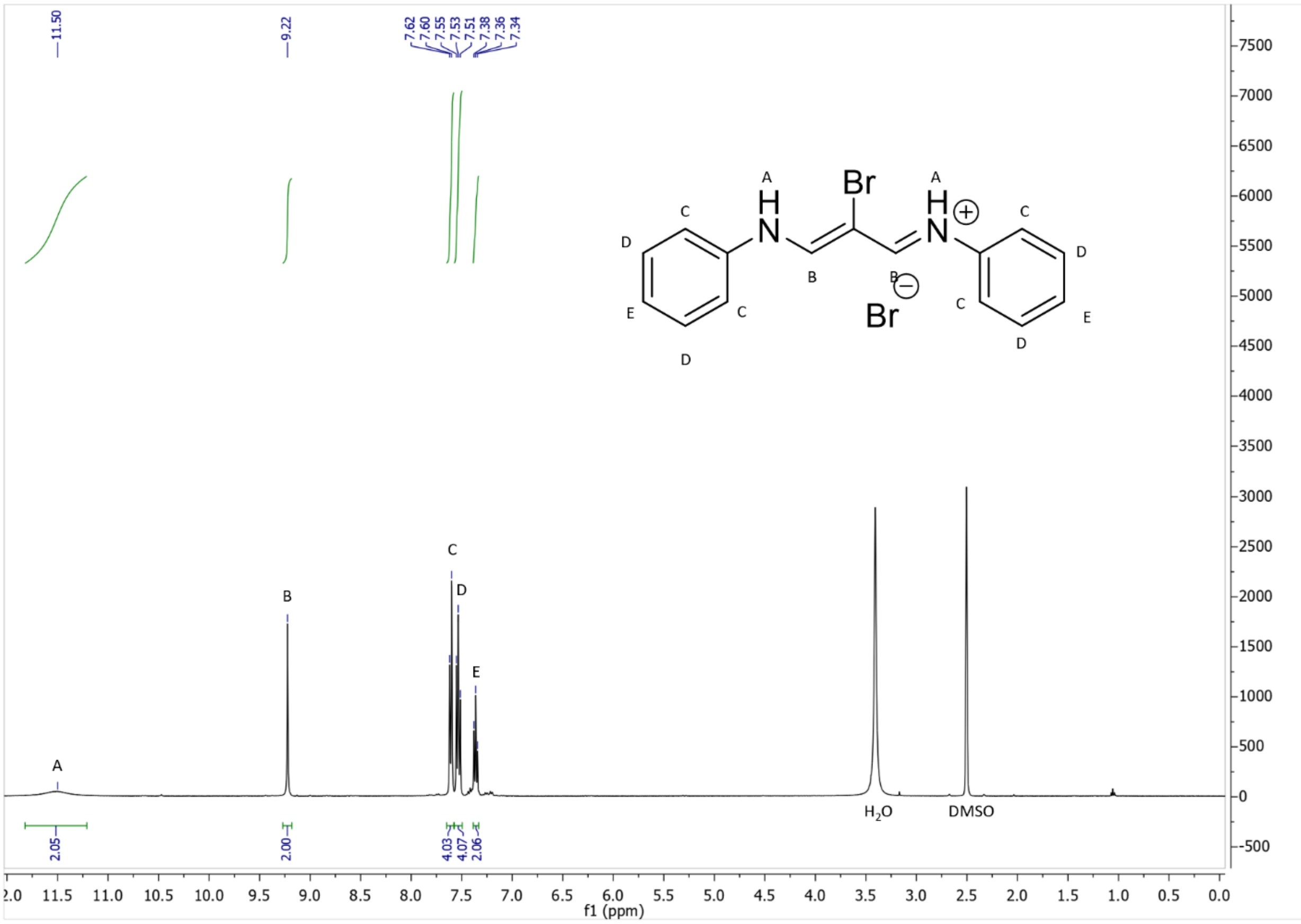

^{1}H NMR spectrum (400 MHz, DMSO-d6, 25°C) of **S1**.

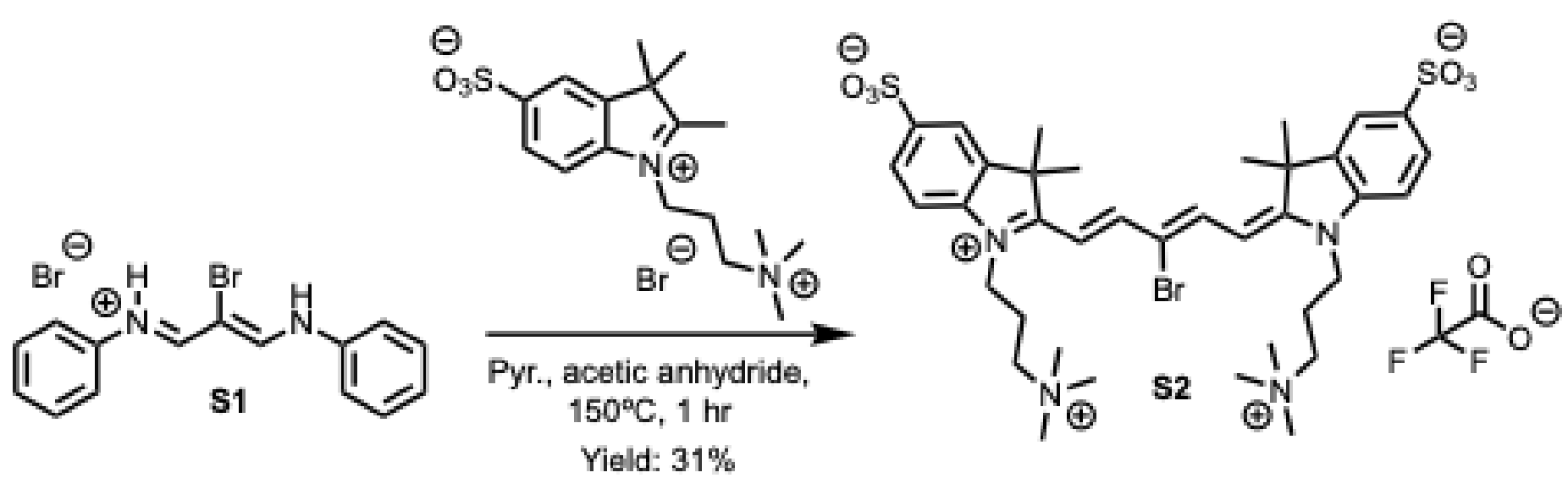

Scheme S3. Synthesis of **S2**.

S2: Prepared by a known method.[2,3] 2,3,3-trimethyl-1-(3-(trimethylammonio)propyl)-3H-indol-1-ium-5-sulfonate bromide (0.3564 g) and **S1** (0.1478 g) were dissolved in 2.1 mL of acetic anhydride and then 0.1 mL of pyridine was added. The mixture was heated to 150 °C for 1 hour. The reaction mixture was dissolved in about 10 mL of methanol and then 40 mL of diethyl ether was added to precipitate the crude dye. The mixture was centrifuged and then washed with about 50 mL of diethyl ether. The crude product was then purified using reverse phase flash chromatography using 0-30% methanol (0.5% TFA)/water as the eluent to yield **S2** as a blue solid (0.1051 g, 31.2%). ^{1}H NMR (400 MHz, DMSO-d6, 25 °C) δ 8.61 (d, 2H), 7.93 (s, 2H), 7.64 (d, 2H), 7.51 (d, 2H), 6.37 (d, 2H), 4.26 (t, 4H), 3.54 (t, 4H), 3.09 (s, 18H), 2.21 (p, 4H), 1.76 (s, 12H).

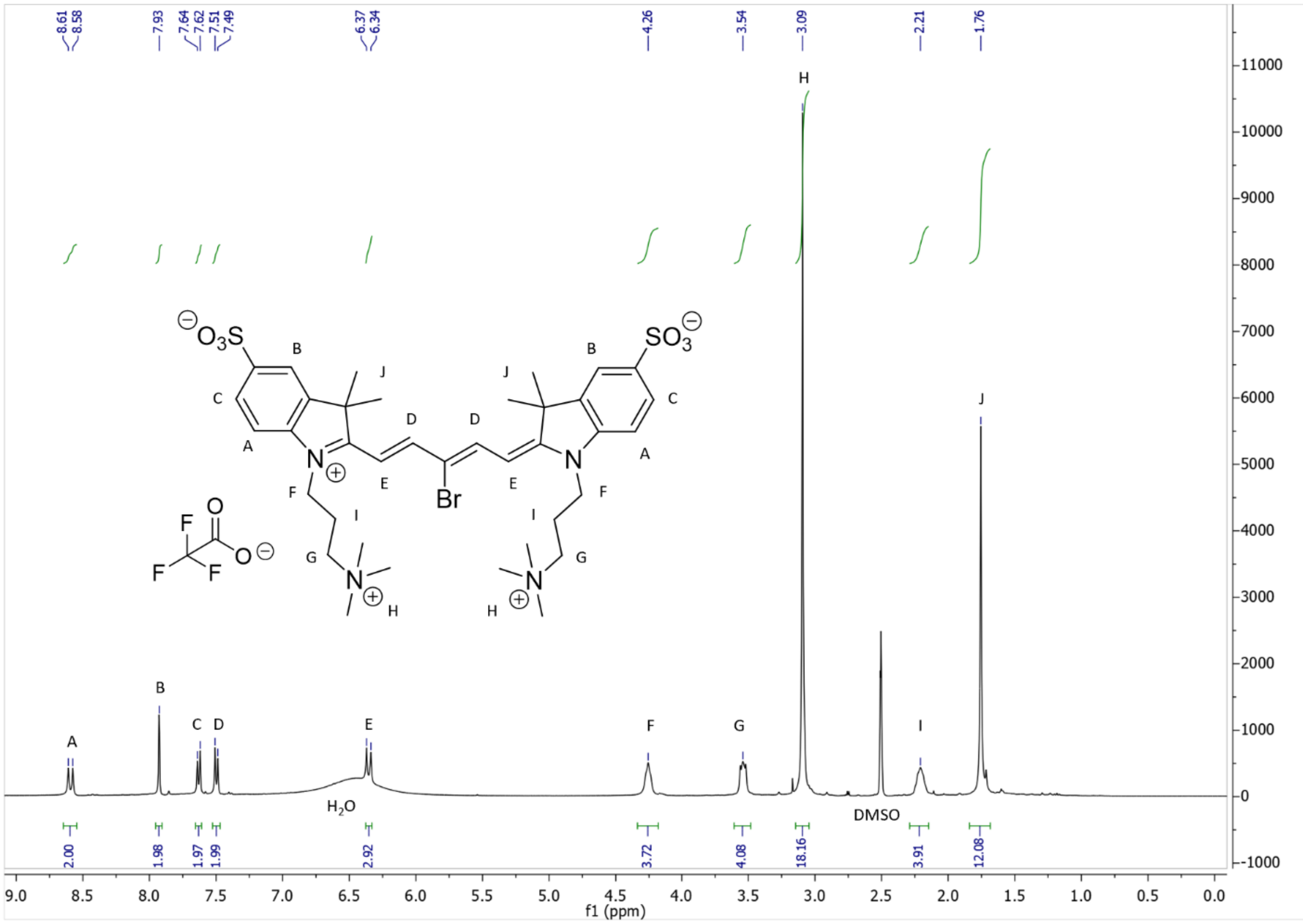

^{1}H NMR spectrum (400 MHz, DMSO-d6, 25°C) of **S2**.

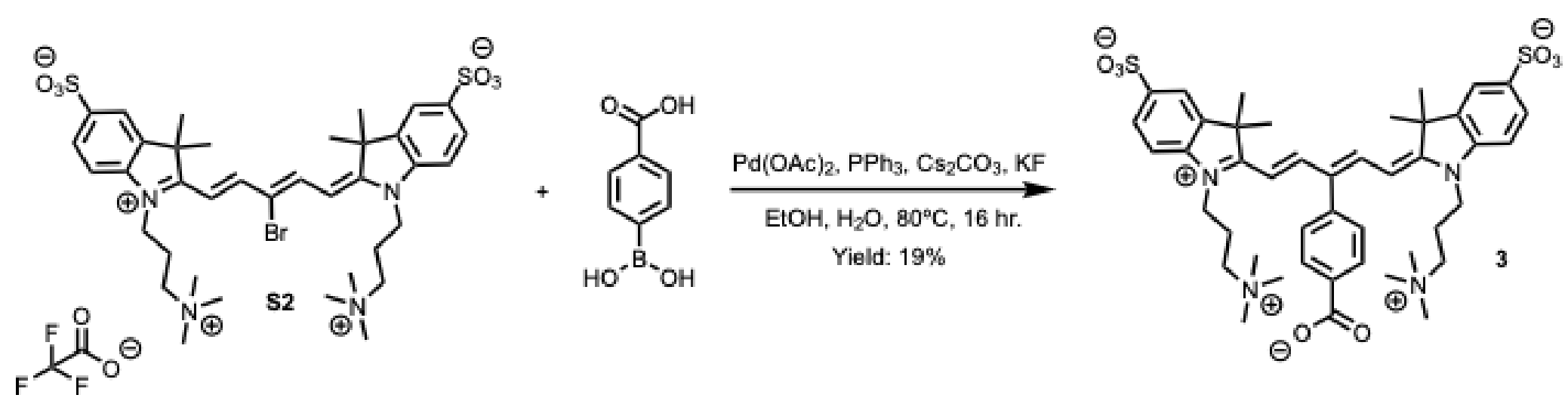

Scheme S4. Synthesis of known **Control dye 3**.[3]

Control dye 3: Prepared by a known method.[3] Intermediate **S2** (114.1 mg, 0.1259 mmol, 1 eq.), 4-boronobenzoic acid (42.09 mg), potassium fluoride (17.66 mg, 0.3022 mmol, 2.4 eq.), and cesium carbonate (99.21 mg, 0.3022 mmol, 2.4 eq.) were dissolved in a mixture of 4 mL of ethanol and 4 mL of water. Palladium acetate (2.91 mg, 0.01259 mmol, 0.1 eq), and triphenylphosphine (44.43 mg, 0.04406 mmol, 0.35 eq.) were then added to the reaction mixture, which was subsequently heated to 85 °C for 17 hrs. The solvents were then evaporated under reduced pressure. The mixture was then dissolved in water and filtered through celite. The filtrate was purified using reverse phase flash chromatography using 0-40% methanol (with 0.5% TFA) and water to yield control dye **3** as a blue solid (20.2 mg, 19.2%). ^{1}H NMR (400 MHz, DMSO-d6, 25 °C) δ 8.58 (d, 2H), 8.13 (d, 2H), 7.88 (s, 2H), 7.61 (d, 2H), 7.51 (d, 2H), 7.37 (d, 2H), 5.65 (d, 2H), 3.85 (t, 4H), 3.24 (t, 4H), 3.00 (s, 18H), 1.95 (p, 4H), 1.79 (s, 12H).

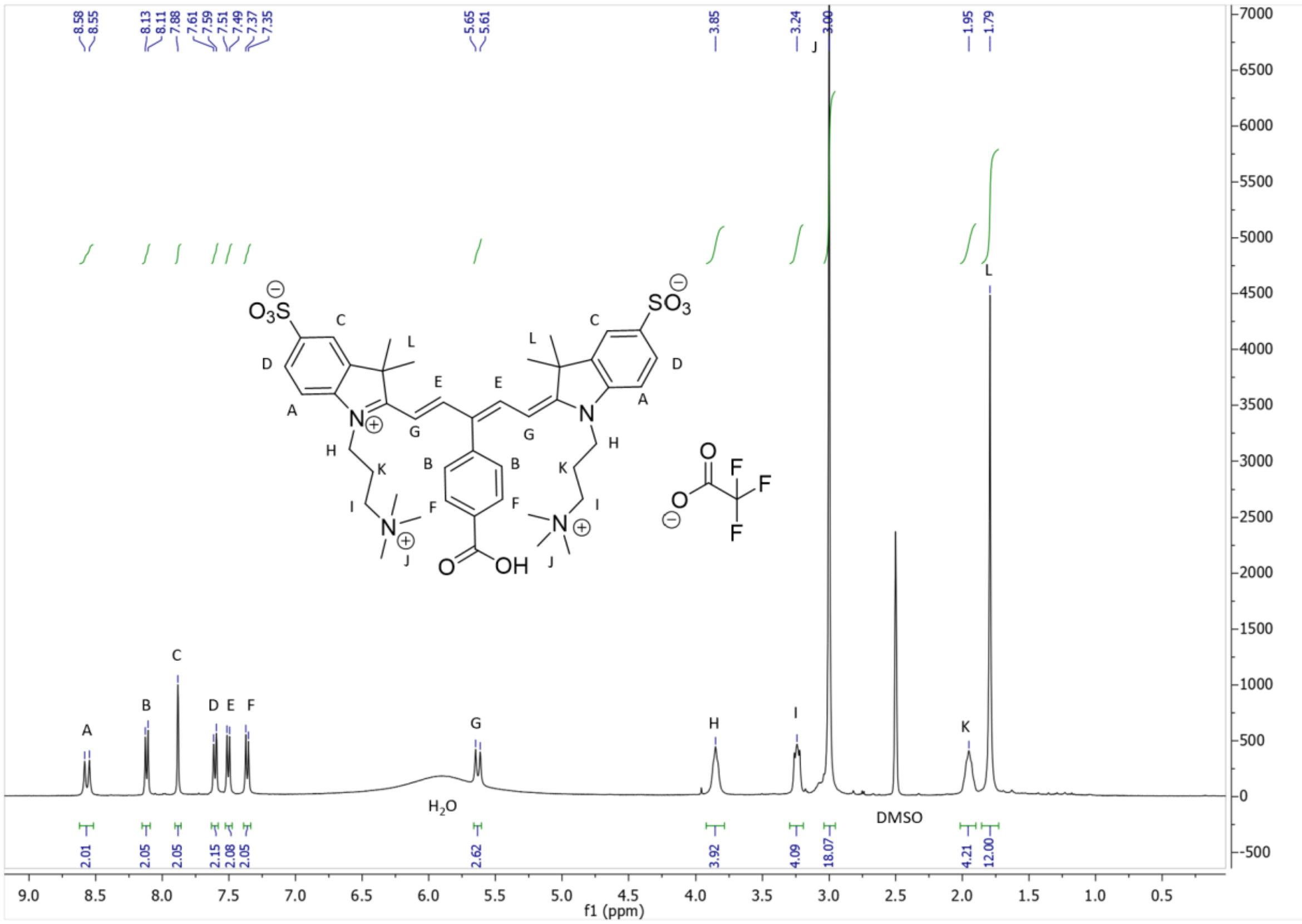

^{1}H NMR spectrum (400 MHz, DMSO-d6, 25°C) of known **Control dye 3**.

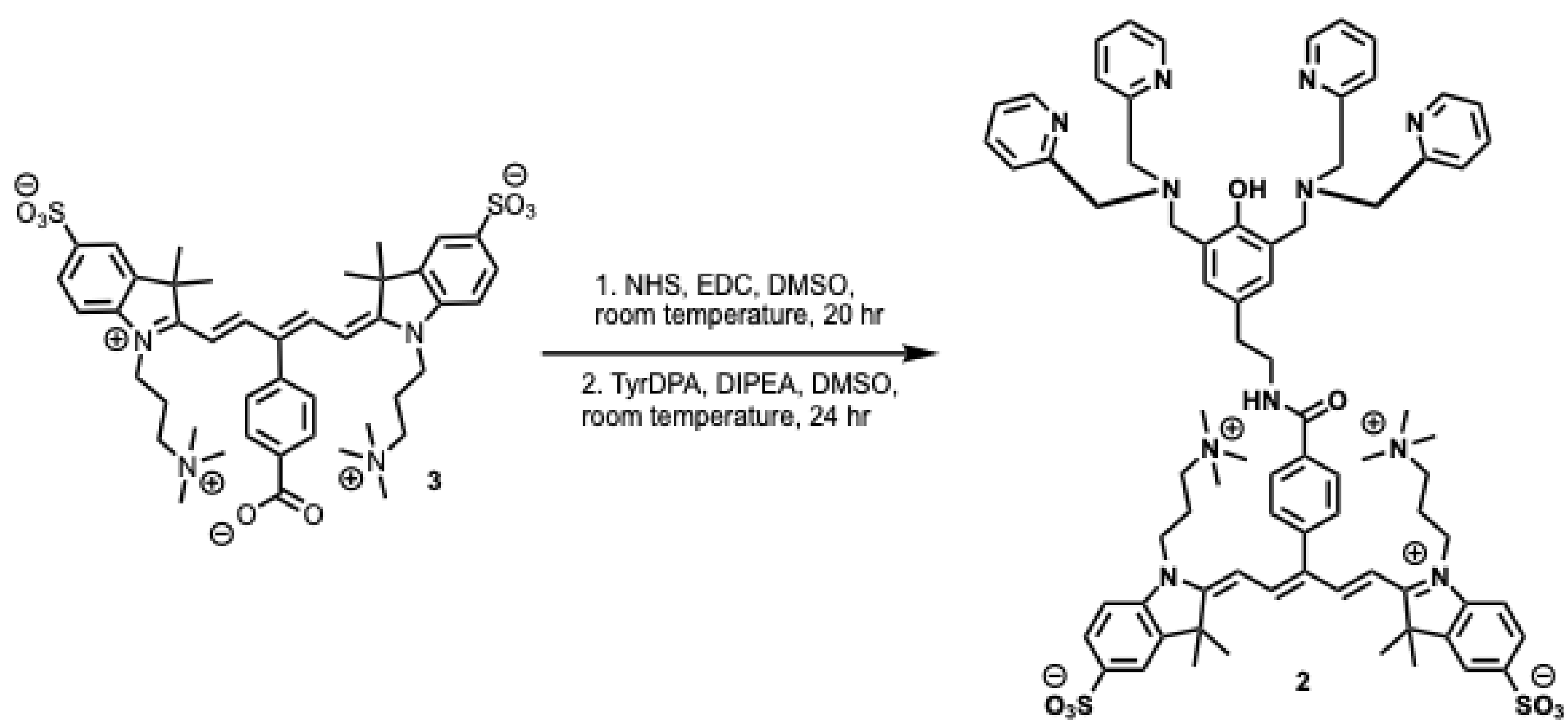

Scheme S5. Synthesis of apo-receptor **2**

Control dye **3** (18.0 mg, 0.0190 mmol, 1 eq.), N-Hydroxysuccinimide (8.81 mg, 0.0765 mmol, 3.5 eq.), and 1-Ethyl-3-(3-dimethylaminopropyl)carbodiimide (13.22 mg, 0.0689 mmol, 3.2 eq.) were dissolved in 2 mL of anhydrous DMSO and stirred at room temperature under an argon atmosphere protected from light for 19.5 hours. 40 mL of ethyl acetate was added to precipitate the NHS ester from solution. This solid was again washed with 40 mL of ethyl acetate and finally with 40 mL of diethyl ether to afford the NHS ester of control dye **3** as a dark blue solid. Without further purification this material (approximately 0.02 mmol, 1 eq.) was dissolved in 1 mL of DMSO and mixed with a solution of TyramineBDPA[3] (0.0258 g, 0.0432 mmol, 2 eq.) in 1 mL of DMSO. The combined DMSO solutions were treated with 18.8 µL of DIPEA. The mixture was stirred at room temperature for 23 hours under an argon atmosphere protected from light. The reaction mixture was purified directly using reverse phase chromatography with 20-50% methanol (0.5% TFA). The resulting product was washed with two 40 mL portions of diethyl ether and dried under vacuum to afford apo-receptor **2** as an indigo colored solid (16.3 mg, 58% yield). Apo-receptor **2** was treated with two molar equivalents of $Zn(NO_3)_2$ in methanol and stirred at room temperature for 30 min. The solvent was evaporated to obtain receptor **2**. ^1H NMR (400 MHz, DMSO-d6, 25 °C) δ 8.93 (t, 1H), 8.58 (m, 6H), 8.08 (d, 2H), 7.89 (s, 2H), 7.84 (t, 4H), 7.63 (d, 2H), 7.48-7.36 (m, 12H), 7.16 (s, 2H), 5.66 (d, 2H), 4.26 (s, 8H), 4.16 (s, 4H), 3.85 (t, 4H), 3.46 (m, 2H), 3.21 (t, 4H), 2.99 (s, 18H), 2.74 (m, 2H), 1.95 (p, 4H), 1.79 (s, 12H). HRMS (ESI+): calculated for $C_{78}H_{93}N_{11}O_8S_2$ $[M+H]^{2+}$ 687.8320, found 687.8339.

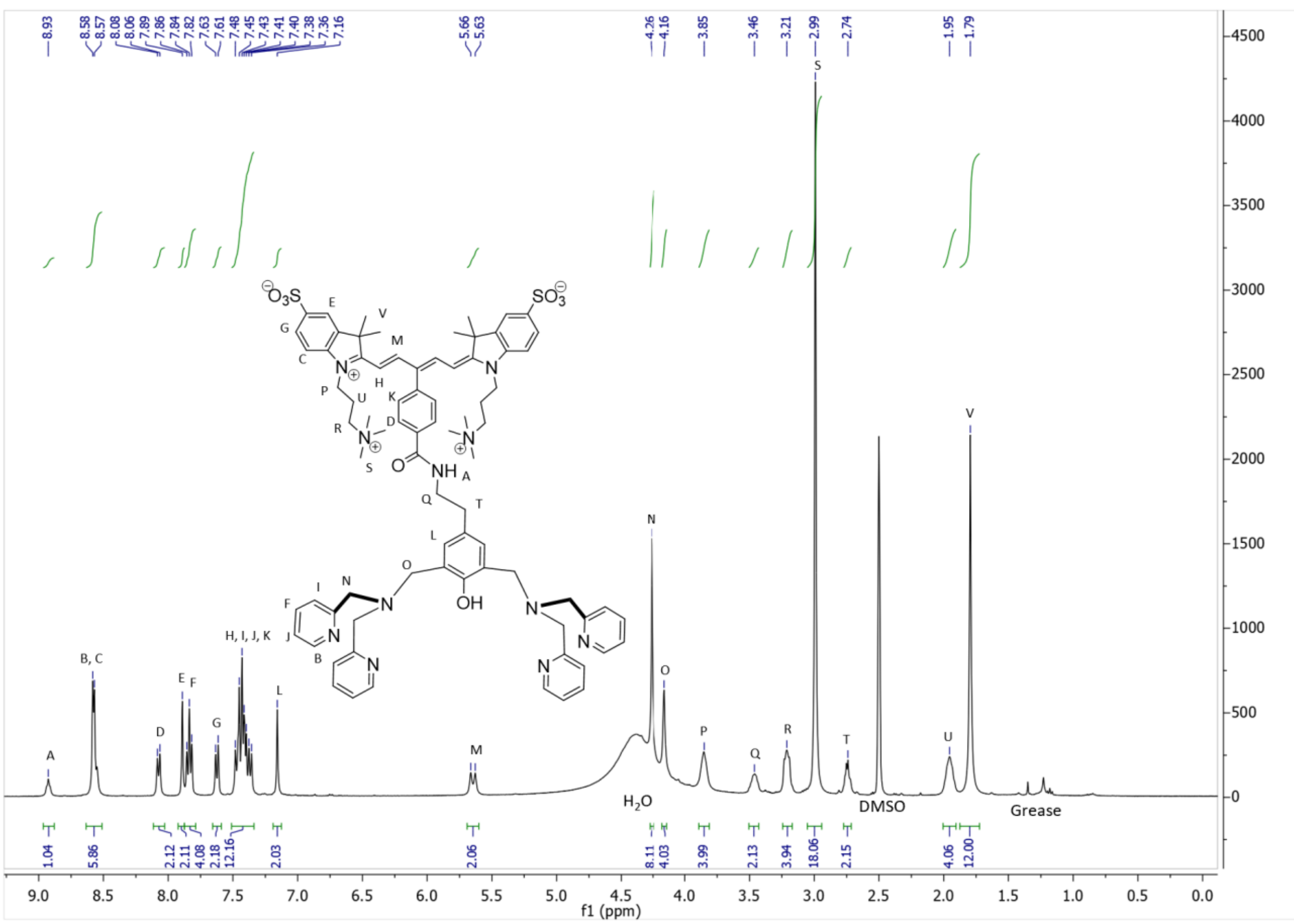

^1H NMR spectrum (400 MHz, DMSO-d6, 25°C) of apo-receptor **2**

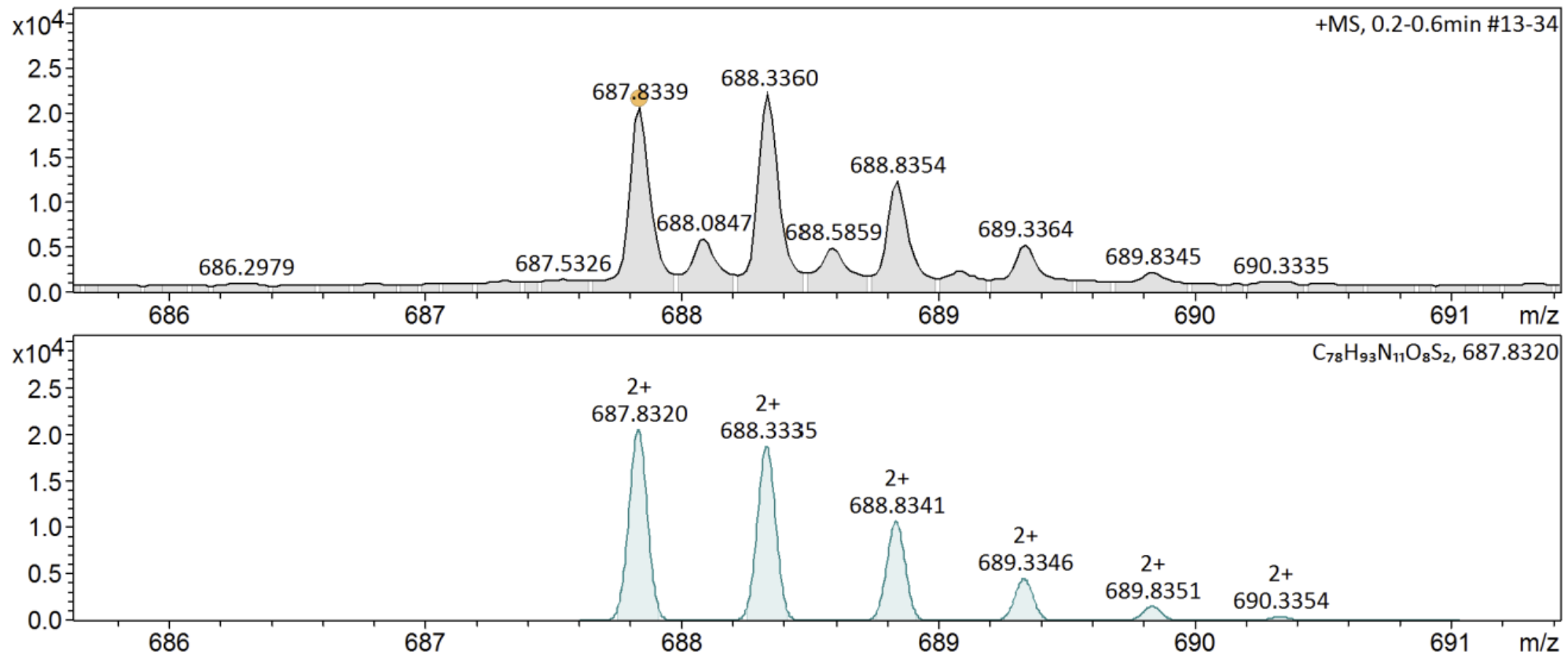

Meas. m/z	#	Ion Formula	m/z	err [ppm]	Mean err [ppm]	rdb	N-Rule	e⁻ Conf	mSigma	Std I	Std Mean m/z	Std I VarNorm	Std m/z Diff	Std Comb Dev
687.833920	1	C78H93N11O8S2	687.831952	-2.9	-1.9	38.0	ok	even	53.2	47.6	n.a.	n.a.	n.a.	n.a.

Mass spectrum of apo-receptor **2**

3. Spectral Data for Receptor 2 and Control Dye 3

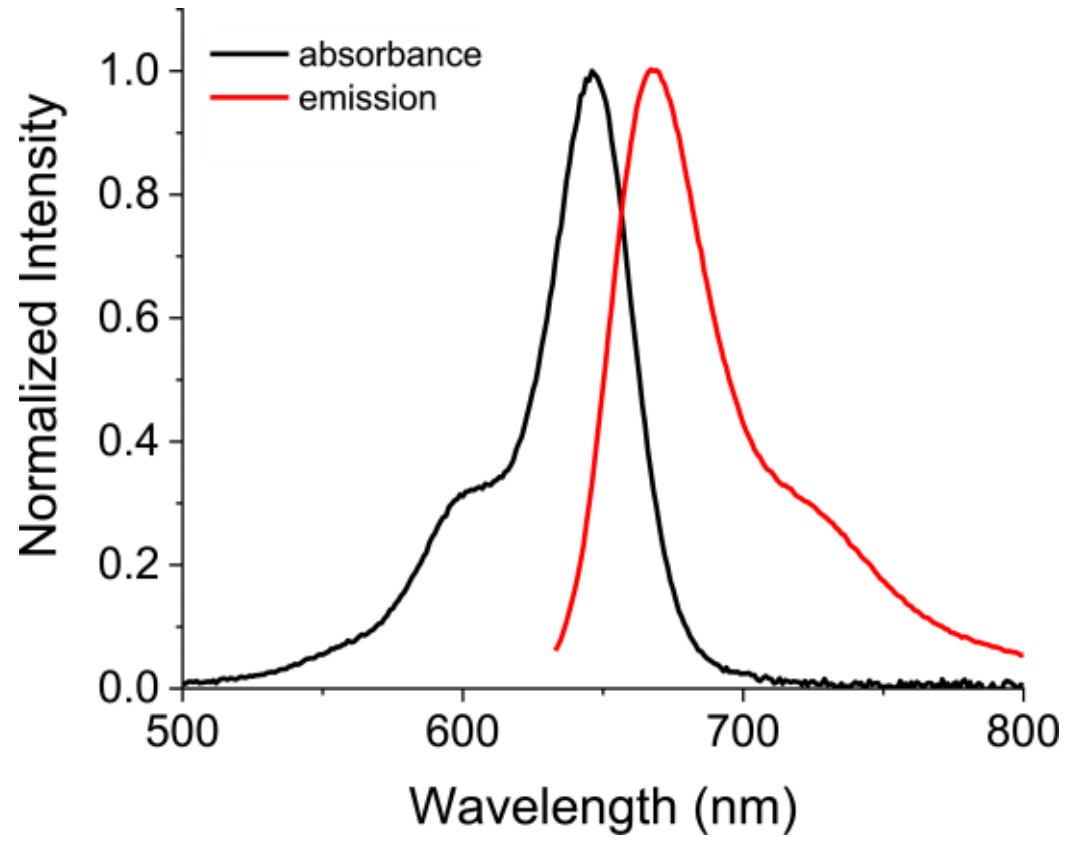

Figure S1. Normalized intensity of receptor **2** (5 μM) in 100% MeOH (λ_{ex}: 623 nm, slit width: 3 nm).

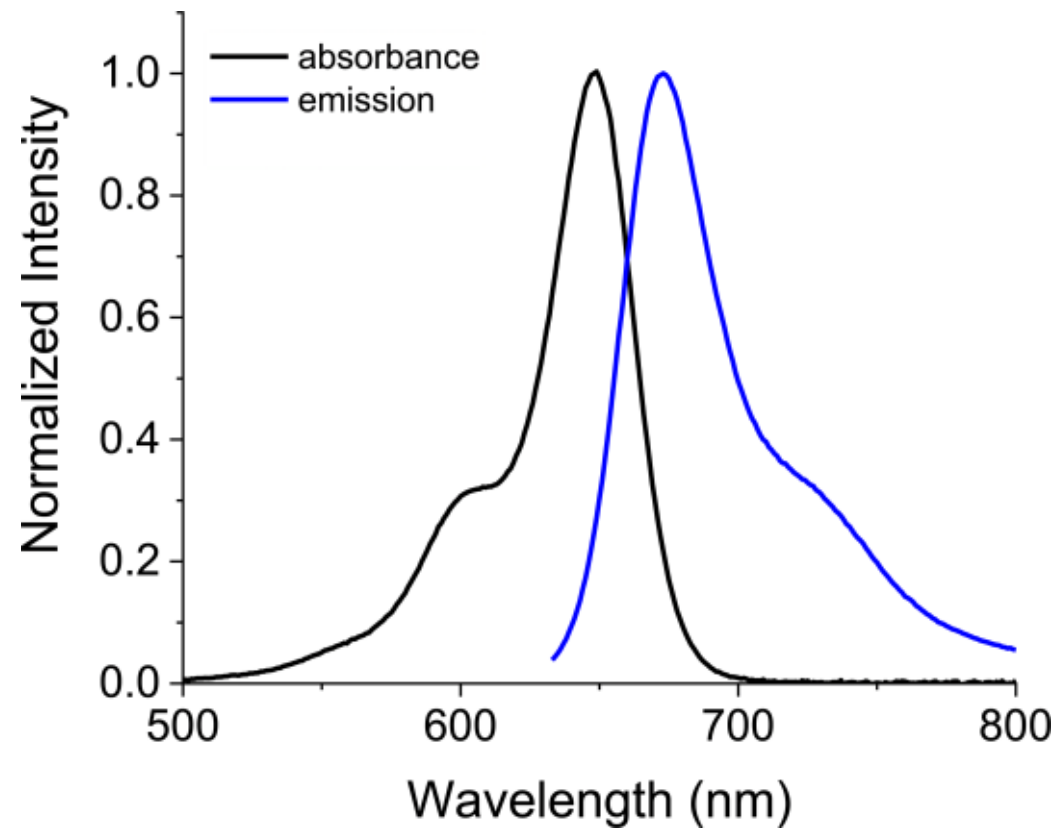

Figure S2. Normalized intensity of control dye **3** (5 μM) in 100% MeOH (λ_{ex}: 623 nm, slit width: 3 nm).

Table S1. Spectral data for receptor **2** and control dye **3** in HEPES buffer (10 mM, pH 7.2).

Dye	λ_{max}^{abs} (nm)	λ_{max}^{em} (nm)	ε ($M^{-1}cm^{-1}$)	ϕ_F [%][a]
Receptor **2**	643	665	115,000	6.6 ± 0.5
Control dye **3**	643	665	130,000	7.4 ± 0.2

[a] Relative to indocyanine blue in HEPES buffer (10 mM, pH 7.2).[4]

4. Pyrocatechol Violet Displacement Studies

The assay solution was prepared by mixing pyrocatechol violet (PV) and receptor **1** in a 1:1 molar ratio (each 20 μM) in an aqueous solution of HEPES buffer (10 mM, pH 7.2), resulting in the formation of a blue colored **1**:PV complex. Anion displacement of PV from the **1**:PV complex resulted in a visible color change from blue to yellow (**Scheme S6**). Absorbance spectra were collected by scanning over the range 800-350 nm and the absorbance change at λ_{max} = 631 nm was plotted to give titration isotherms.

The assay solution was prepared by mixing pyrocatechol violet (PV) and receptor **1** in a 1:1 molar ratio (each 20 μM) in an aqueous solution of HEPES buffer (10 mM, pH 7.2) to a total volume of 1 mL, resulting in the formation of a blue colored **1**:PV complex. Anion displacement of PV from the **1**:PV complex resulted in a visible color change from blue to yellow (**Scheme S6**). Absorbance spectra were acquired over the range 800-350 nm and the absorbance change at λ_{max} = 631 nm was plotted to give titration isotherms.

Anions were added as aliquots from 8 mM stock solutions in HEPES buffer (10 mM, pH 7.2) containing **1**:PV (20 μM, to ensure unchanged **1**:PV concentrations throughout the titration). Anion concentrations of 0-200 μM were obtained by sequential addition of 1.25 μL aliquots until 100 μM. followed by 6.25 μL aliquots to obtain the 150 and 200 μM data points. The final solution volume after completion was 1.025 mL. After each addition the solution was inverted six times before the absorbance spectrum was obtained.

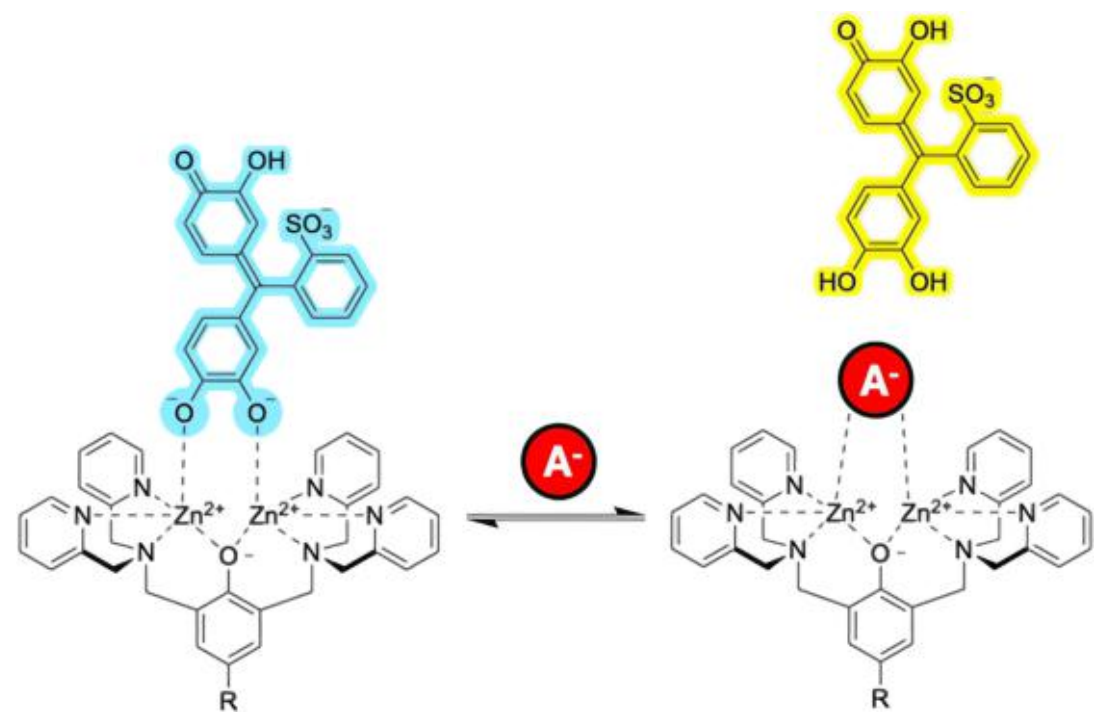

Scheme S6. Anions displace the Pyrocatechol Violet (PV) from receptor **1** and lower the PV absorbance at 631 nm.

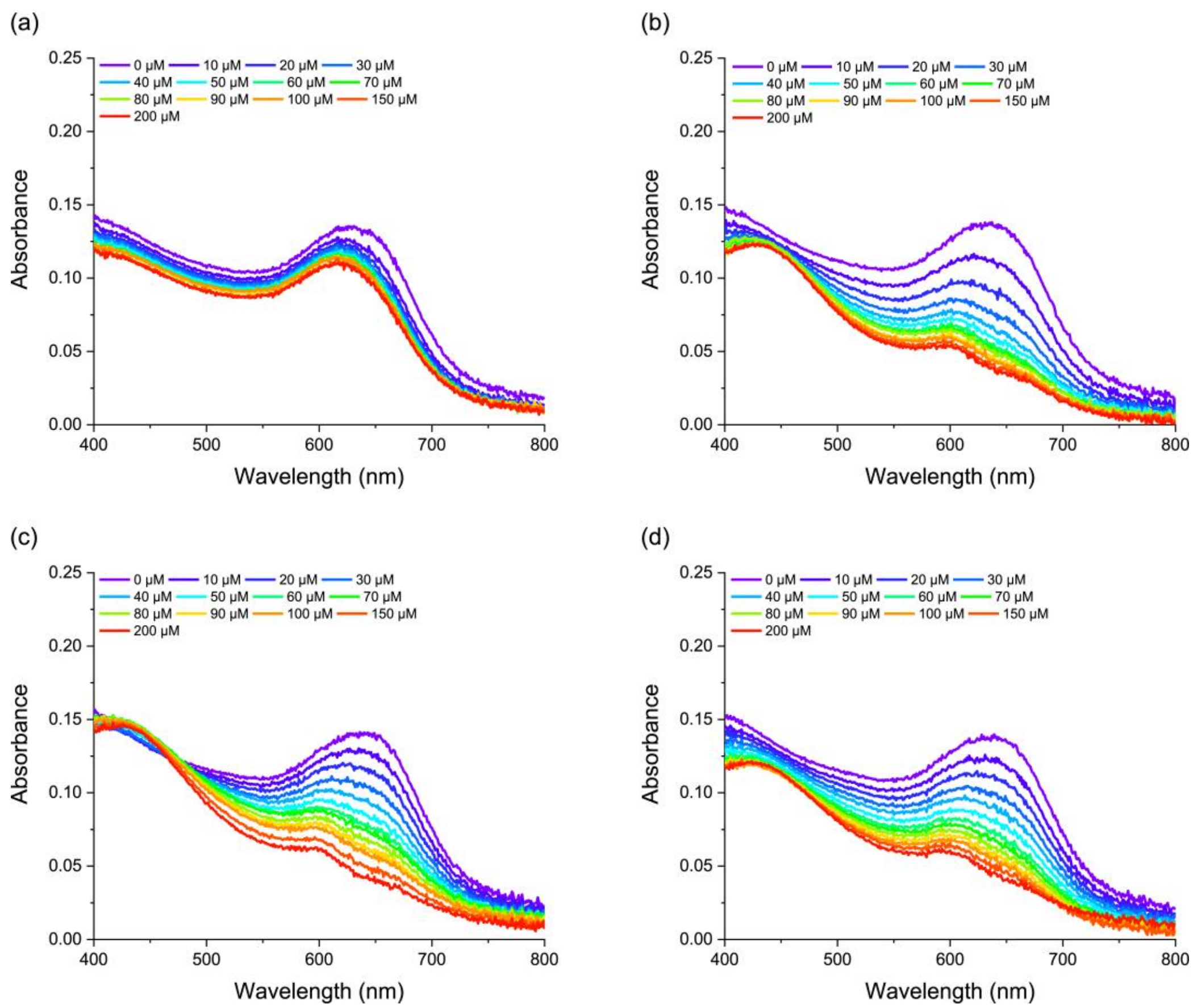

Figure S3. Representative absorbance spectra from the colorimetric pyrocatechol violet (PV) displacement assay for a binary mixture of receptor **1** and PV (each 20 μM) upon addition of carboxylate anions (a) sodium acetate, (b) sodium citrate, (c) sodium polyacrylate, or (d) sodium alginate in HEPES buffer (10 mM, pH 7.2). All measurements were performed at room temperature in triplicate.

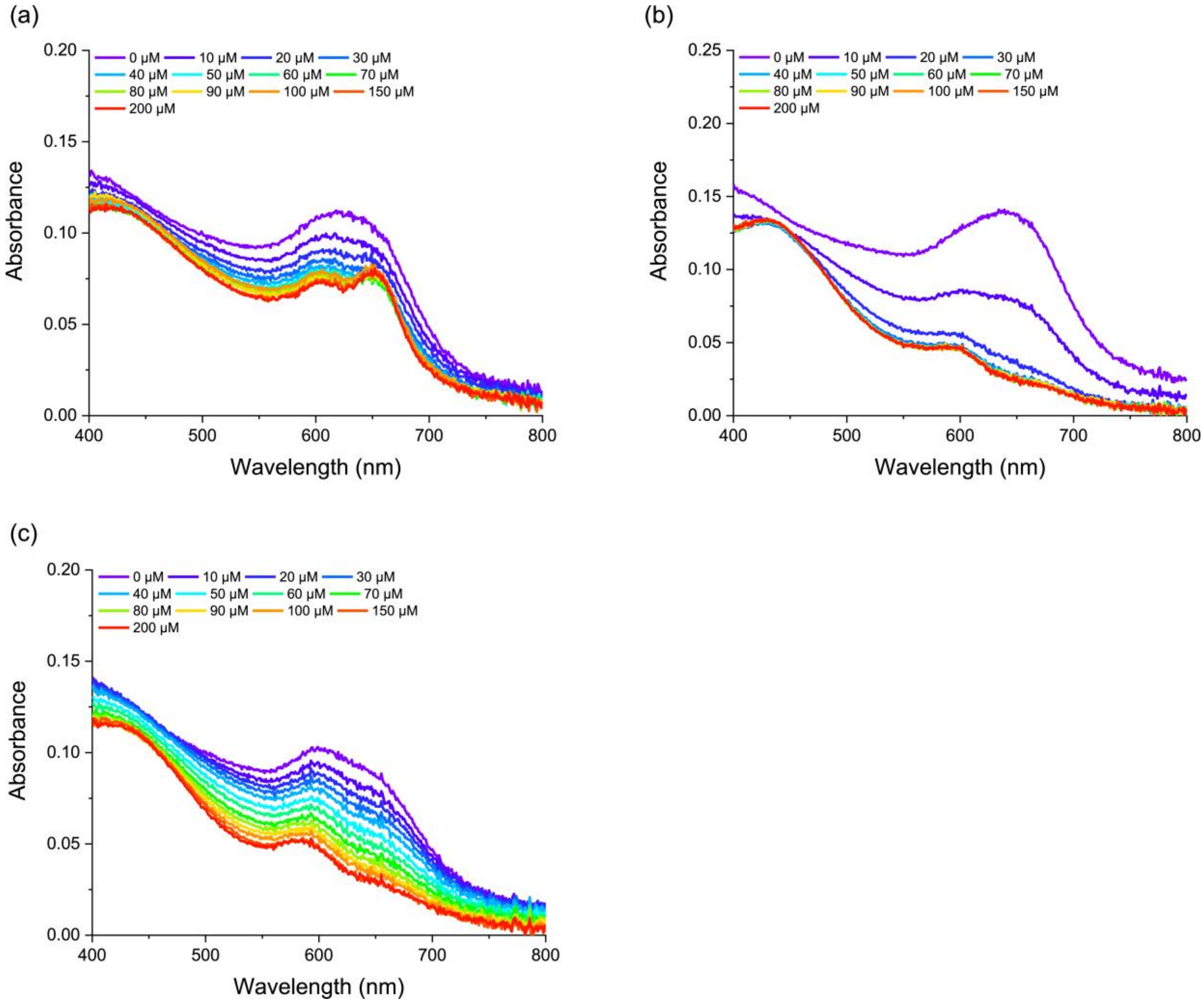

Figure S4. Representative absorbance spectra from the colorimetric pyrocatechol violet (PV) displacement assay for a binary mixture of receptor **1** and PV (each 20 μM) upon addition of phosphate anions (a) sodium phosphate, (b) sodium pyrophosphate, or (c) sodium polyphosphate in HEPES buffer (10 mM, pH 7.2). All measurements were performed at room temperature in triplicate.

Polysaccharide Polyanion Binding Measured Using Pyrocatechol Violet Displacement Assay

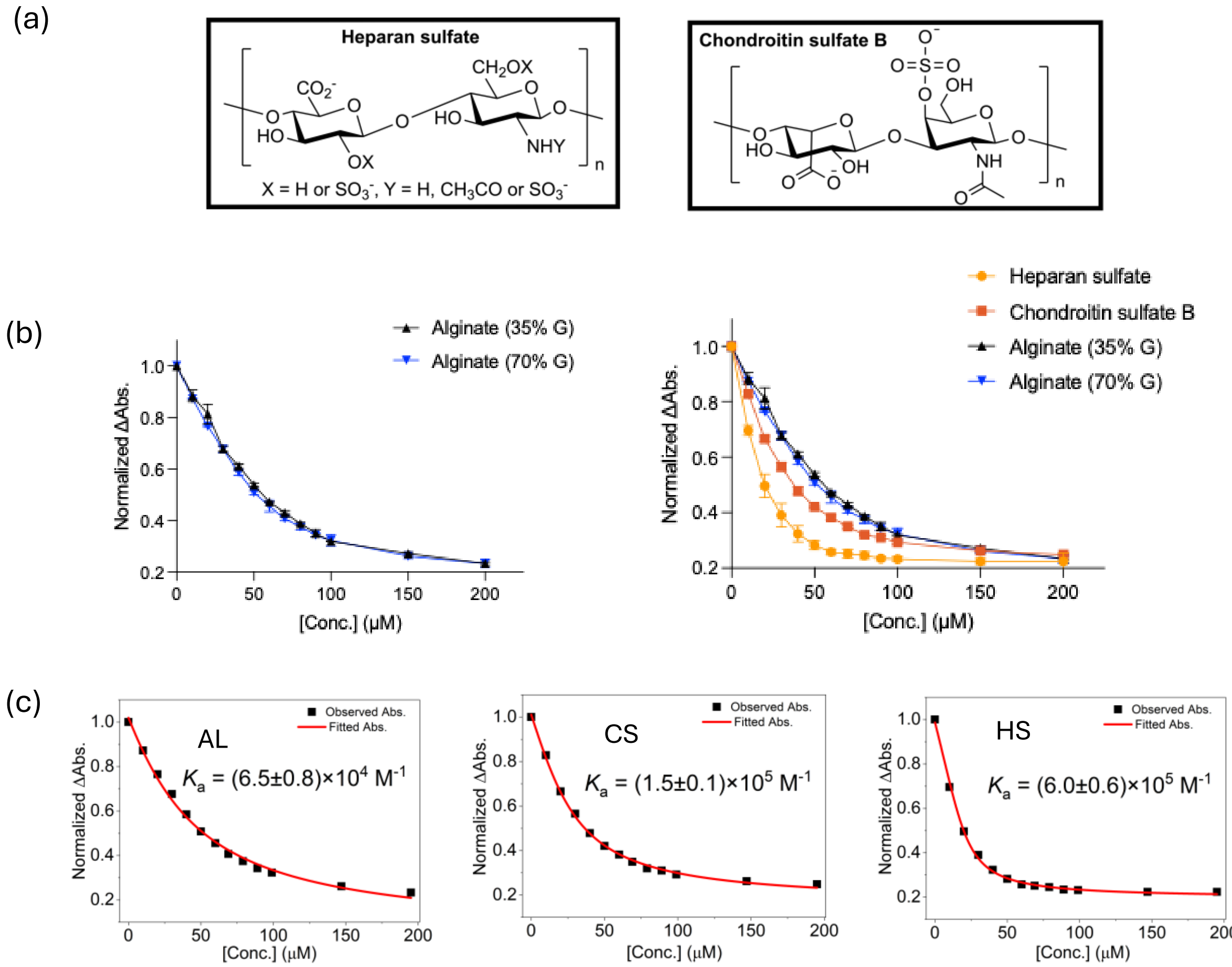

Figure S5. Titrations using the colorimetric Pyrocatechol Violet (PV) displacement assay. (a) Structures of the added polyanions. (b) Plots showing change in absorbance at 631 nm for a binary mixture of **1** and PV (each 20 μM) in HEPES buffer (10 mM, pH 7.2) upon addition of sodium alginate with 70% G content (Sigma Cat. No. 71238), sodium alginate with 35% G content (Sigma Cat. No. A2033), sodium chondroitin sulfate B (Sigma Cat. No. C3788) or heparan sulfate (MedChem Express Cat. No. HY-101916). All measurements were performed at room temperature in triplicate. (c) Non-linear fitting of titration isotherms to the indicator displacement model described below and the derived value of K_a for receptor **1** binding to alginate (AL), chondroitin sulfate B (CS), or heparan sulfate (HS).

Indicator Displacement Method for Determining the Anion Binding Constant

Assume two independent 1:1 binding equilibrium between the host **1** (H), PV indicator (I), and anion (A):

$$\mathrm{H + I \rightleftharpoons HI} \qquad K_I = \frac{[\mathrm{HI}]}{[\mathrm{H}][\mathrm{I}]}$$

$$\mathrm{H + A \rightleftharpoons HA} \qquad K_A = \frac{[\mathrm{HA}]}{[\mathrm{H}][\mathrm{A}]}$$

1. Exact Competitive Binding Model

Define

- $H_0 = [H]_{tot}$
- $I_0 = [I]_{tot}$
- $A_0 = [A]_{tot}$
- h = [H] (free host concentration)

From the mass balances,

$$[\mathrm{HI}] = \frac{K_I h I_0}{1+K_I \mathrm{h}}$$

$$[\mathrm{HA}] = \frac{K_A h A_0}{1+K_A \mathrm{h}}$$

The host mass balance is therefore:

$$H_0 = h + \frac{K_I h I_0}{1+K_I \mathrm{h}} + \frac{K_A h A_0}{1+K_A \mathrm{h}} \quad (1)$$

For each anion concentration A_0, solve Eq. (1) for h. The predicted concentration of the host–indicator complex is

$$[\mathrm{HI}] = \frac{K_I h I_0}{1+K_I \mathrm{h}} \quad (2)$$

2. Simplification for $H_0 = I_0 = C = 20\ \mu M$

Equation 1 becomes

$$\mathrm{C} = h + \frac{K_I h C}{1+K_I \mathrm{h}} + \frac{K_A h A_0}{1+K_A \mathrm{h}} \quad (3)$$

After clearing the denominators, the free-host concentration is the physical root of the cubic equation

$$K_A K_I h^3 + (A_0 K_A K_I + K_A + K_I)h^2 + (A_0 K_A + 1)h - C = 0 \quad (4)$$

Once *h* is obtained,

$$[HI] = \frac{K_I h C}{1+K_I h} \qquad (5)$$

The physical root must satisfy

$$0<h<C$$

3. Signal Equation

Because the measured absorbance or fluorescence is linear, use

$$Y = Y_\infty + S[HI] \quad (6)$$

Where:

- Y_∞ is the signal when the indicator is completely displaced;
- *S* is an instrumental scaling factor;
- K_A is the anion binding constant to be fitted;
- K_I should normally be fixed at the independently measured indicator-binding constant.

A more convenient normalized expression is

$$Y = Y_\infty + (Y_0 - Y_\infty)\frac{[HI]}{[HI]_0} \quad (7)$$

where $[HI]_0$ is the complex concentration before adding anion.

For $H_0 = I_0 = C$

$$[HI]_0 = \frac{2C+\frac{1}{K_I}-\sqrt{(2C+\frac{1}{K_I})^2-4C^2}}{2} \quad (8)$$

Therefore, the fitting parameters can be limited to

K_A, Y_0 and Y_∞

Where K_I, H_0 and I_0 are fixed.

Origin C Implementation

The most numerically stable approach is to solve Equation 1 within an Origin C nonlinear fitting function using Newton's iterative method (A. E. Hargrove, Z. Zhong, J. L. Sessler and E. V. Anslyn *New J. Chem.*, 2010, **34**, 348–354).

The Newton–Raphson equation is

$$f(h) = \mathrm{h} + \frac{K_I h I_0}{1 + K_I \mathrm{h}} + \frac{K_A h A_0}{1 + K_A \mathrm{h}} - H_0$$

With derivative

$$f'(h) = 1 + \frac{K_I I_0}{(1 + K_I \mathrm{h})^2} + \frac{K_A A_0}{(1 + K_A \mathrm{h})^2}$$

The following script was used for fitting using the OriginLab software

```
// x = total anion concentration A0
// y0 = signal before anion addition
// yinf = signal after complete displacement
// Ka = anion-host association constant
// Ki = indicator-host association constant
// H0 = total host concentration
// I0 = total indicator concentration

double h;
double f;
double df;
double hnew;
double HI;
double HI0;
double disc;
int i;

// Initial estimate of free host
h = H0 / (1.0 + Ki*I0 + Ka*x);

if (h <= 0.0)
   h = H0 * 0.01;

// Solve the host mass-balance equation
for (i = 0; i < 100; i++)
{
   f = h
       + Ki*h*I0/(1.0 + Ki*h)
       + Ka*h*x/(1.0 + Ka*h)
       - H0;

   df = 1.0
```

```
        + Ki*I0/((1.0 + Ki*h)*(1.0 + Ki*h))
        + Ka*x/((1.0 + Ka*h)*(1.0 + Ka*h));

    hnew = h - f/df;

    // Keep the root within the physically meaningful range
    if (hnew <= 0.0)
        hnew = h/2.0;

    if (hnew >= H0)
        hnew = (h + H0)/2.0;

    if (fabs(hnew - h) < 1e-12)
        break;

    h = hnew;
}

// Host-indicator complex at anion concentration x
HI = Ki*h*I0/(1.0 + Ki*h);

// Host-indicator complex before anion addition
disc = (H0 + I0 + 1.0/Ki)*(H0 + I0 + 1.0/Ki)
       - 4.0*H0*I0;

HI0 = 0.5*((H0 + I0 + 1.0/Ki) - sqrt(disc));

// Predicted experimental signal

y = yinf + (y0 - yinf)*(HI/HI0);
```

Parameter Settings

Parameter	Setting
K_A	Fit
K_I	44500 M^{-1}
H_0	0.00002 M
I_0	0.00002 M
Y_0	Fit
Y_∞	Fit

5. Alginate Microsphere Studies

Preparation of Preloaded Microspheres

An aqueous solution of 1.0% (w/v) sodium alginate containing either no additive, receptor **2** (100 μM), or control dye **3** (100 μM) was added dropwise to a 7.5 mL aqueous solution of 0.5% (w/v) $CaCl_2$ in a 50 mL centrifuge tube. The drops were delivered at room temperature using a 21-gauge hypodermic needle (inner diameter = 0.514 ± 0.019 mm) positioned 5 cm above the solution. The empty or preloaded Ca-crosslinked alginate microspheres formed immediately and were collected within 2 minutes by filtration and washed with deionized water. This simple hydrogel fabrication method was quite reproducible, and the average microsphere diameter was 2.30 ± 0.17 mm.

Leakage of Receptor 2 or Control Dye 3 from Preloaded Microspheres

Ten Ca-crosslinked alginate microspheres, preloaded with either receptor **2** or control dye **3**, were transferred to a 4 mL quartz cuvette containing 3 mL of HEPES buffer (10 mM, pH 7.2). In the case of the enhanced leakage experiments, the external solution contained 1 mM of additive (pyrophosphate, NaCl, receptor **1,** or polyphosphate) in HEPES buffer (10 mM, pH 7.2). At each 10-minute time point over a total period of 4 hours, the microspheres were quickly removed from the external solution, the external solution absorbance at 643 nm was measured, and the microspheres returned to the external solution. Between each absorbance measurement, the cuvette was gently inverted to ensure complete solution mixing. The absorbance values were plotted to give the 2-hour leakage profiles in **Figure 3**. Leakage of receptor **2** after 24 hours is summarized below in **Figure S6**.

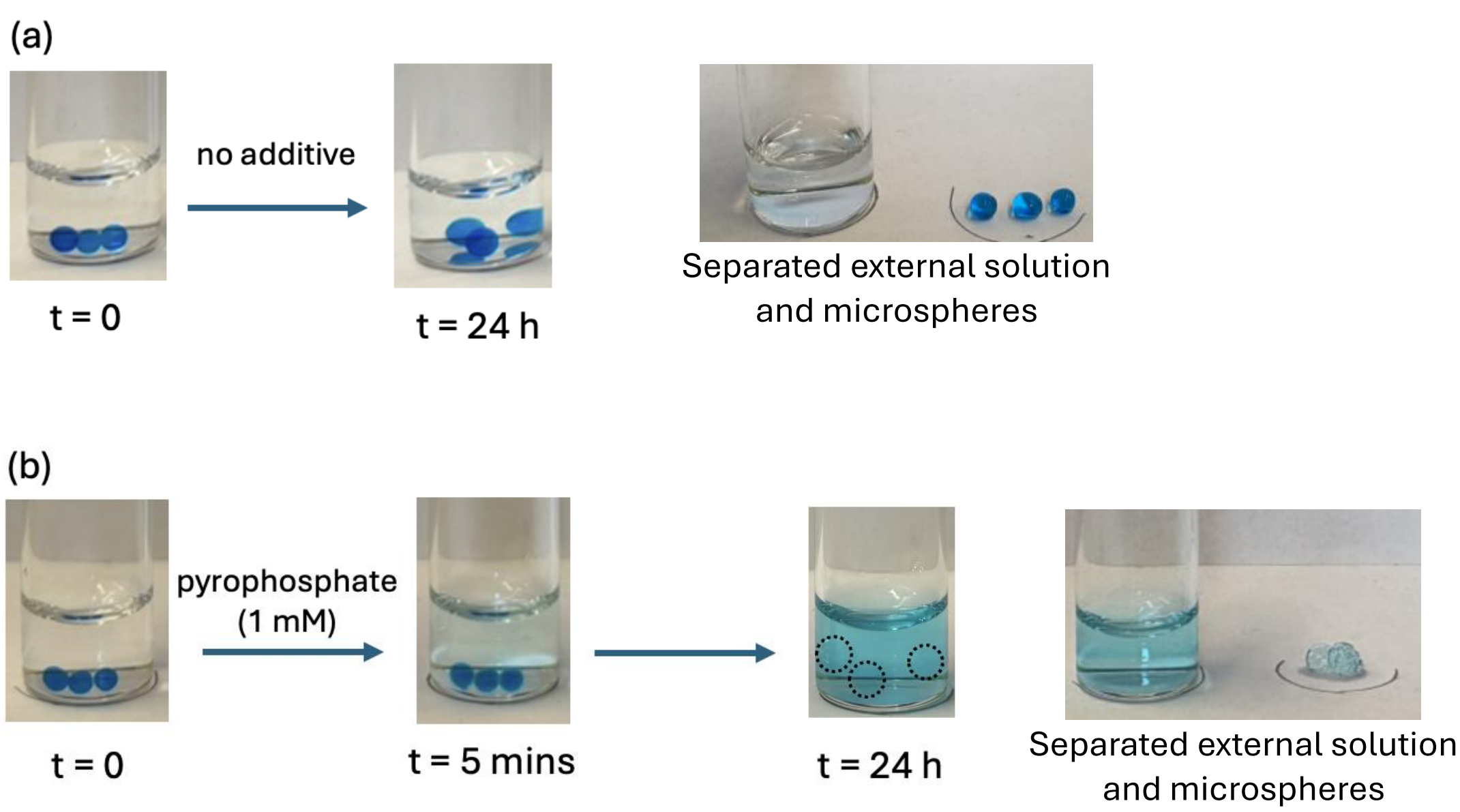

Figure S6. Photographs showing extent of receptor **2** leakage from three Ca-crosslinked alginate microspheres that were incubated for 24 hours in an external solution of: (a) HEPES buffer (10 mM, pH 7.2), or (b) sodium pyrophosphate (1 mM) in HEPES buffer (10 mM, pH 7.2). The presence of pyrophosphate induces complete displacement of blue-colored **2** from the alginate strands after 24 hours and it is equally dispersed throughout the microspheres and external solution.

Calcium Leaching from Ca-crosslinked Alginate Hydrogel Microspheres

Ten empty Ca-crosslinked alginate microspheres were prepared as described above and submerged for 2 hours in an external solution (1 mL) of (i) HEPES buffer (10 mM, pH 7.2), (ii) HEPES buffer + 1 mM polyphosphate, or (iii) HEPES buffer + 1 mM pyrophosphate. The external solutions were removed, and the calcium content was determined using a RayBiotech Calcium Assay Kit (Colorimetric) (MA-CA-1). In this assay, calcium reacts with the indicator dye Arsenazo in a slightly alkaline medium to form a purple-colored complex whose absorbance at 650 nm is proportional to the calcium content in the sample. As shown in **Figure S7a**, the calcium concentration in all three external solutions was statistically equal. That is, all three external solutions induced essentially the same level of Ca-leaching from the ten microspheres, and each solution reached the same equilibrium Ca-concentration. Additionally, a photograph of the separated microspheres following the incubations (**Figure S7b**) indicates no discernible morphological changes.

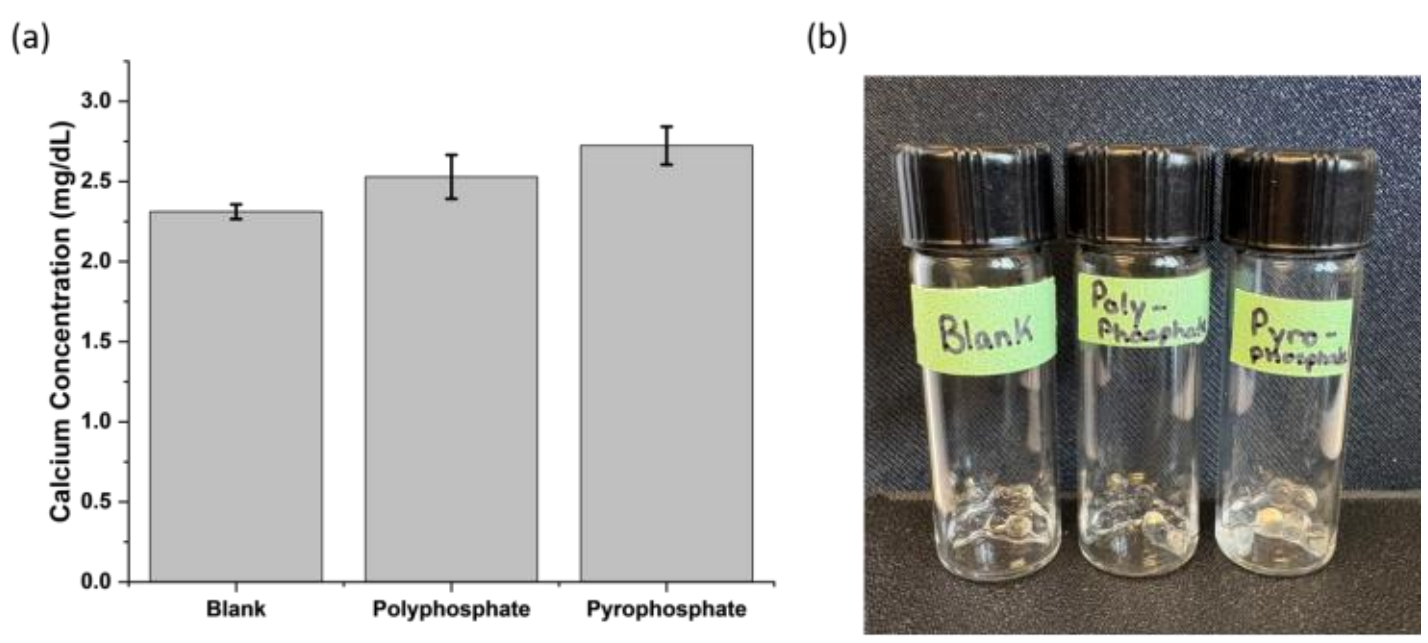

Figure S7. (a) Calcium concentration in external solutions after 2-hour incubation of 10 Ca-crosslinked microspheres in (i) HEPES buffer (10 mM, pH 7.2), (ii) HEPES buffer + 1 mM polyphosphate, or (iii) HEPES buffer + 1 mM pyrophosphate and incubated for 2 hours. The bar graph reports the average of three experiments ± SE. All three external solutions exhibited the same level of Ca-leaching from the ten microspheres and reached statistically the same equilibrium Ca-concentration. (b) Photograph of the separated microspheres following incubation indicates no discernible morphological changes.

Loading into Empty Ca-crosslinked Alginate Hydrogel Microspheres

Absorption spectroscopy method: Ten empty Ca-crosslinked alginate microspheres were submerged in an external solution (1 mL) of either receptor **2** or control dye **3** (100 µM in each case) and incubated for 24 hours (N = 3). The microspheres were removed and the concentration of **2** or **3** in the external solution was determined from the absorbance at 643 nm.

Fluorescence imaging method using Eppendorf tube: A single, empty Ca-crosslinked alginate microsphere was placed in a 0.25 mL Eppendorf tube (N = 3) containing 40 µL receptor **2** or control dye **3** (10 µM in each case) in HEPES buffer (10 mM, pH 7.2). After a 24-hour incubation, the microsphere was removed and imaged alongside the Eppendorf tube containing the external solution (**Figure 5**). The Eppendorf tube images in **Figure S8** include several shorter incubation periods.

Fluorescence imaging method using multiwell array: Forty-eight microwells in a 96-microwell plate were charged with a single, empty Ca-crosslinked alginate microsphere and an external solution (200 µL) of receptor **2** or control dye **3** (10 µM) in HEPES buffer (10 mM, pH 7.4). After 24-hour incubation, each microsphere was removed and placed in adjacent microwell, and the entire 96-microwell plate was imaged (**Figure S8**).

For both imaging methods, brightfield and fluorescence images were acquired using a small animal in vivo station (Spectral instruments, AMI HT) with the following settings: excitation 605/20 nm, emission 670/20 nm, exposure 3 s, power 50%, F-stop 2, FOV 20, binning: low. The digital images were analyzed using ImageJ software. A region-of-interest (ROI) was drawn around the separated external solution and microsphere, and in each case the Mean Pixel Intensity (MPI) was determined.

Alginate Microsphere Loading Studies: Fluorescence Imaging of Microsphere Loading Experiments in Eppendorf Tubes

This low throughput protocol produced three data sets for each condition to give the graph in **Figure S8b**.

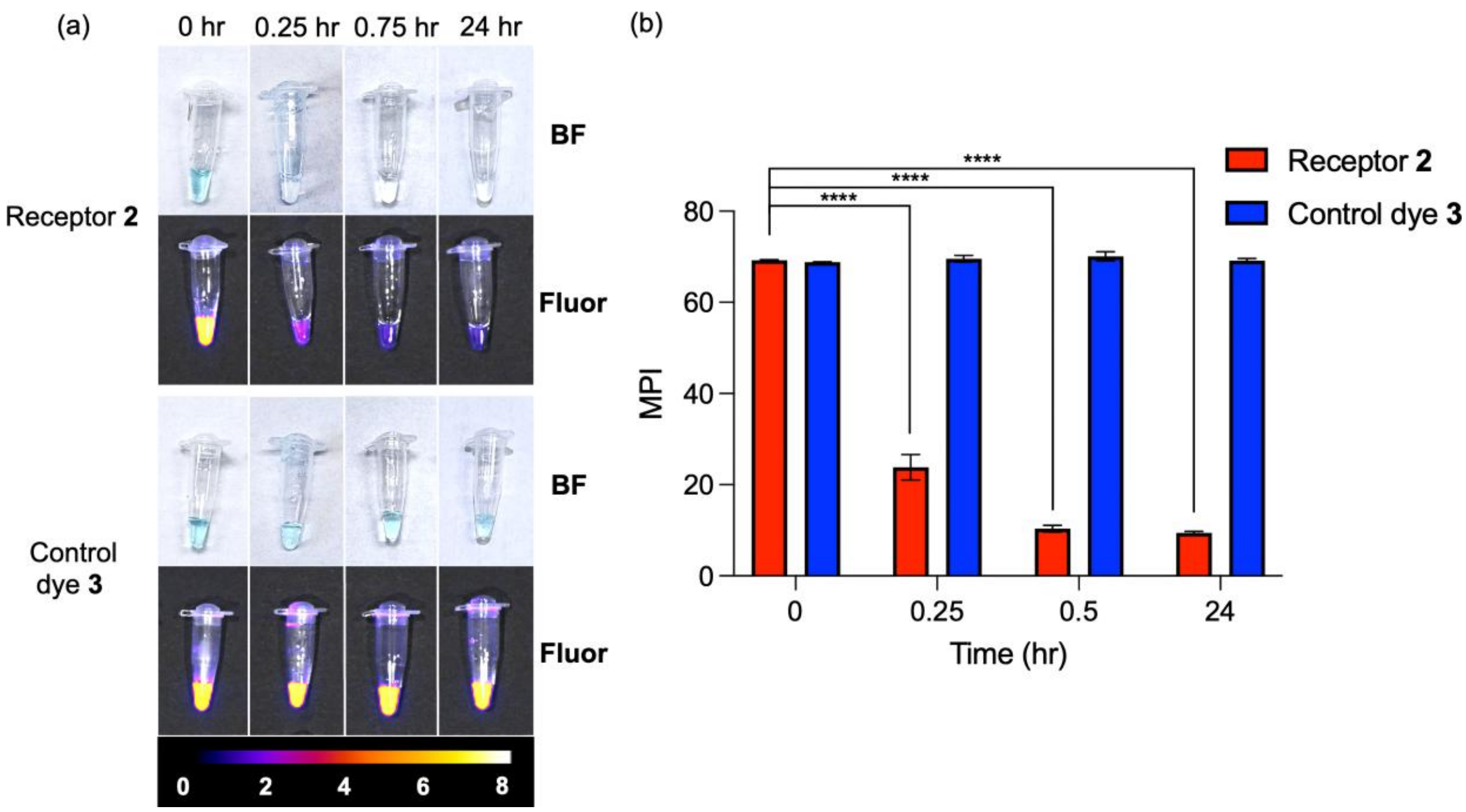

Figure S8. (a) Representative brightfield and fluorescence images of Eppendorf tubes containing solutions of receptor **2** or control dye **3** (10 μM in HEPES buffer, pH 7.2) before adding a single microsphere (t = 0 hr) or after incubation for a designated time point (t = 0.25, 0.5, 24 hr) and microsphere removal. (b) Fluorescence mean pixel intensity (MPI) for the Eppendorf tubes containing a solution of **2** or **3** before adding a single microsphere (t = 0 hr) or after microsphere incubation for a designated time point and microsphere removal (t = 0.25, 0.5, 24 hr). All fluorescence images were obtained using an in vivo imaging station (ex: 605 nm, em: 670 nm) and each experiment was conducted in triplicate. Error bars show ± SEM, **** $p < 0.0001$.

Alginate Microsphere Loading Studies: Fluorescence Imaging of Microsphere Loading Experiments in a Multiwell Array

This high throughput protocol produced twenty-four data sets for each condition to give the graph in **Figure S9c** which is consistent with the graph in article **Figure 5c**.

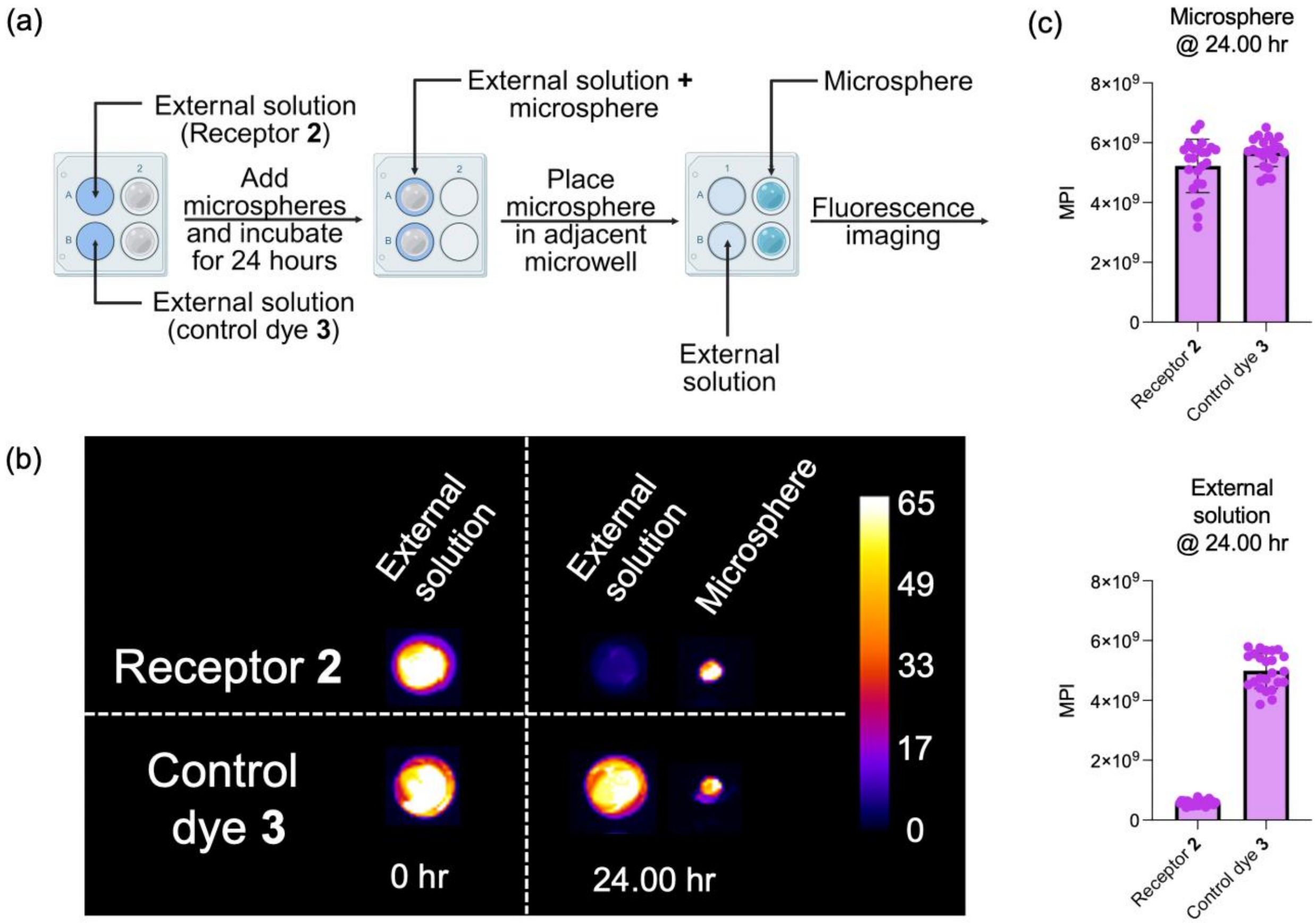

Figure S9. (a) Schematic representation of two concurrent microsphere loading experiments based on fluorescence imaging using a multiwell plate. Briefly, fluorescence images were acquired for a microwell containing a solution of **2** (10 µM in HEPES 10 mM, pH 7.4) and a separate microwell containing a solution of **3** (10 µM in HEPES 10 mM, pH 7.4). A single Ca-crosslinked alginate microsphere (2.3 mm in diameter) was placed in each solution. After incubation for 24 hr, the microsphere was removed from each solution and placed in an adjacent empty microwell. Then, a fluorescence image of the four microwells was acquired. (b) Fluorescence images of these two representative microsphere-loading experiments. **Note that the larger 96-microwell plate contained 24 copies of each experiment and thus produced 24 replicates.** (c) Bar graph of mean pixel intensity (MPI) for the fluorescence image of each separated microsphere and corresponding external solution after the 24 hr incubation. Each bar in the graph reflects the 24 replicates for each experiment, all contained within the same microwell array. Fluorescence images acquired using ex: 605 nm, em: 670 nm, with fluorescence intensity scale in arbitrary units.

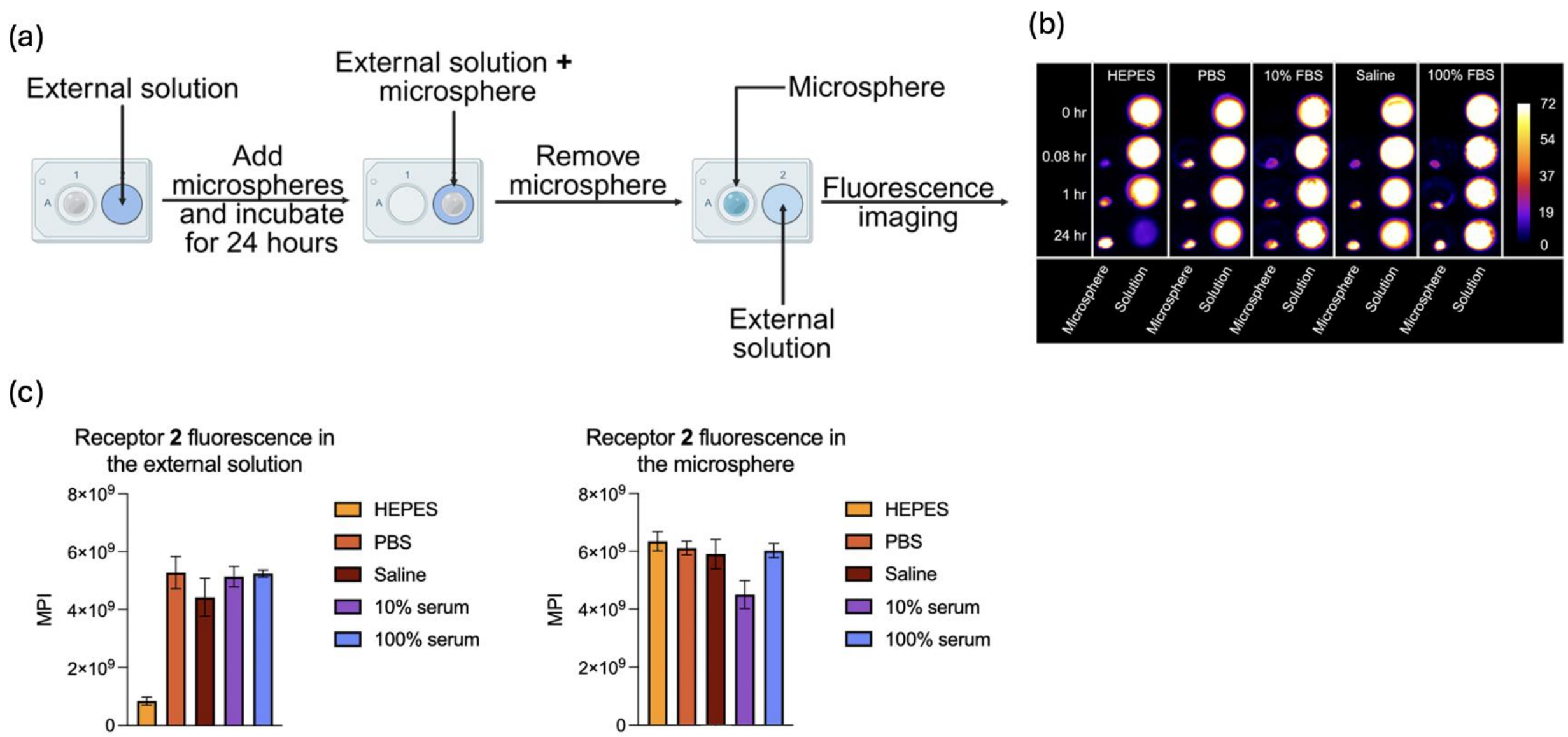

Figure S10. Repeat of the microsphere loading experiment in **Figure S9a** based on fluorescence imaging using a multiwell plate. The only difference between the five experiments in this figure is the composition of the external solution containing receptor **2**; it was either HEPES, PBS, Saline, 10 % FBS, or 100% FBS. (a) Experiment workflow, (b) Fluorescence images at different time points over 24 hours, (c) Quantification of the images after the 24-hour time point. The data shows that after 24 hours there was substantial removal of receptor **2** from the external solution containing only HEPES, and much less removal of receptor **2** when the external solution contained the competitive solutions PBS, Saline, 10 % FBS, or 100% FBS. Nonetheless, the fluorescence signal for each separated microsphere loaded with **2** was about the same for each experiment. Even though the microsphere that was incubated in HEPES contained a much higher amount of receptor **2**, its fluorescence emission was attenuated by self-quenching and inner filter effect. Importantly, loading of **2** from 100% FBS into a Ca-crosslinked alginate microsphere was easily observed by fluorescence imaging and is consistent with the mouse imaging results.

6. Cell Metabolic Activity Measurements

Human pulmonary fibroblasts (HPF) and hepatocellular carcinoma cells (HepG2) were obtained from Promocell GmbH, Germany and ATCC, Manassas, USA respectively. HPF was cultured in Fibroblasts Growth Medium 2 supplemented with vendor provided supplements at 37 °C and 5% CO2 in a humidified incubator. HepG2 was cultured in EMEM medium supplemented with 10% fetal bovine serum and 1% penicillin/streptomycin at 37 °C and 5% CO2 in a humidified incubator. Both cell types were seeded into 96-microwell plates at 5000 cells/well and grown to 80% confluency separately. The medium was then removed and replaced with either receptor **2** or control dye **3** at various micromolar concentrations (N=3, 100 µL) in the respective medium for 24 hr at 37 °C and 5% CO_2 in a humidified incubator. Following the dye incubation, CyQUANT^{TM} XTT Cell Viability Assay Kit (Thermo Fisher Scientific, WA, USA) was used according to the manufacturing recommendation protocol to quantify the cell metabolic activity. The cell metabolic activity of treated cells was plotted relative to a population of untreated cells.

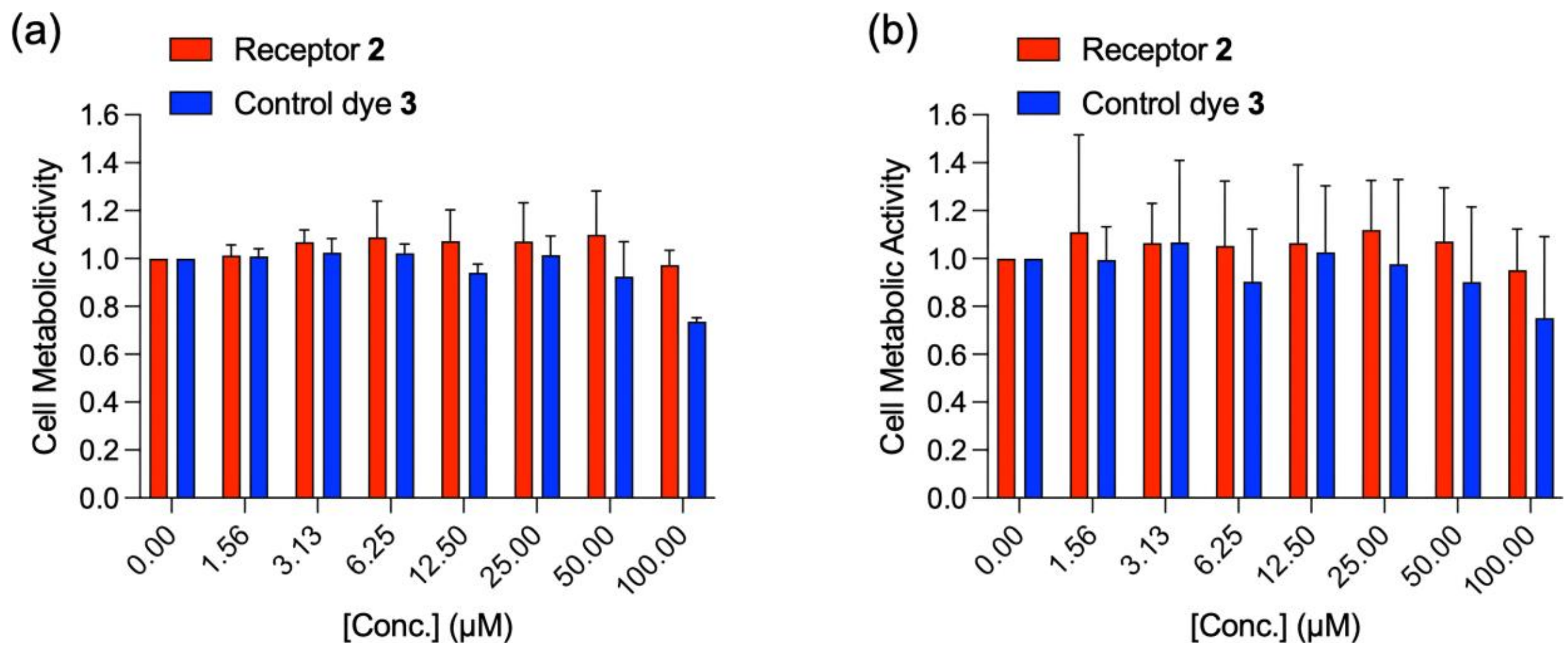

Figure S11. Normalized cell metabolic activity of (a) human pulmonary fibroblasts (HPF) and (b) hepatocellular carcinoma cells (HepG2) following 24 hr exposure to receptor **2** or control dye **3** at biologically relevant concentrations, assessed using an XTT cell metabolic activity assay. Errors bars represent the standard error of the mean from triplicate experiments.

7. Mouse Microsphere Implant Studies

All mouse experiments used female 6–8-week-old athymic nude mice (Foxn1^nu^, strain 553, Charles River Laboratories) and followed protocol 25-09-9572 that was approved by the Notre Dame Institutional Animal Care and Use Committee. The experiments implanted a sterile hydrogel microsphere (4 mm diameter) that was fabricated by dropping a sterile solution of sodium alginate (1.0% w/v, 10 mM HEPES buffer, pH 7.4) solution through a wide-bore P1000 sterile-filtered tip (inner diameter = 1.50 ± 0.02 mm) positioned roughly 5 cm above a sterile $CaCl_2$ solution (0.5% w/v, 10 mM HEPES buffer, pH 7.4). Each microsphere formed immediately and was collected within 2 minutes by filtration and stored in a separate sterile Eppendorf tube. The average microsphere diameter was 4.0 ± 0.1 mm. After the mice reached a body weight >20 g, each mouse was anesthetized using 2–3% isoflurane, and a small subcutaneous pocket was surgically created in the left dorsal area for implantation of a single microsphere. The incision was closed using two 7 mm wound clips, and the mice were allowed to recover with daily observation and care. A palatable gel food supplement was provided in the cage during the recovery period to encourage nutrition until the wound clips were removed after the five-day recovery period. To manage post-operative pain, each mouse received a non-steroidal anti-inflammatory analgesic (meloxicam, up to 5 mg/kg subcutaneously) at least once on the first day after surgery. Additional doses of meloxicam were administered only as needed on subsequent days based on clinical observations of pain or distress. This limited analgesia regimen was chosen to avoid unnecessary prolonged NSAID exposure, which can have minimal added benefit and may contribute to gastrointestinal discomfort or mild weight loss. Body weight was recorded from the day of surgery as a physical indication of post-surgery monitoring and continued until the study endpoint. On day 6, the mice were anesthetized with 5% isoflurane and placed in an in-vivo imaging station to acquire pre-injection fluorescence images using a small-animal imaging system (Spectral Instruments Imaging AMI HT). Each mouse was then injected intravenously with 50 µL of either receptor **2** or control dye **3** at a dose of 100 nmol per mouse via the retro-orbital route (N = 4 for **2**, N = 3 for **3**). Immediately after each injection, the mouse was imaged using the following imaging settings: Far-red filter; ex: 605/20 nm; em: 670/20 nm; exposure: 3 s; percent power: 50%; F-stop: 2; FOV: 20; binning: low. The mice remained under anesthesia (maintained at ~1.5% isoflurane in 1.5 L/min O_2) during imaging to prevent movement. Subsequent live mouse images were acquired at 5 min, 15 min, 30 min, 1 hour, 2 hours, 3 hours, 4 hours, and 24 hours post-injection. Between the longer imaging time points the mice were either kept on a heating pad or transferred to a quarantine housing area where they were continuously monitored. The digital in vivo fluorescence images were analyzed using ImageJ software. A region of interest (ROI) was drawn for target skin covering the implanted microsphere and for background skin located between the neck and the side dorsolateral to the implantation site. The target-to-background ratio (TBR) was determined as the ratio of mean pixel intensity (MPI) values for the two ROIs. At the end of the 24-hour imaging period (implant day 7), the mice were deeply anesthetized with 4% isoflurane and humanely euthanized by cervical dislocation. Immediately following euthanasia, a terminal blood sample was collected via cardiac puncture. The microsphere implant, along with a margin of surrounding skin tissue, was carefully revealed and imaged, then excised along with the major organs (heart, lungs, liver, spleen, kidneys, pancreas, intestines, stomach, muscle, skin surrounding the implant, and skin distal to the implant site). All excised tissues were arranged on a transparent tray and imaged using the AMI HT imaging system and filter settings for in vivo imaging. Using ImageJ software, an ROI was drawn around each harvested organ or tissue, and the MPI was determined and normalized to the MPI of the terminal blood sample obtained from the same mouse.

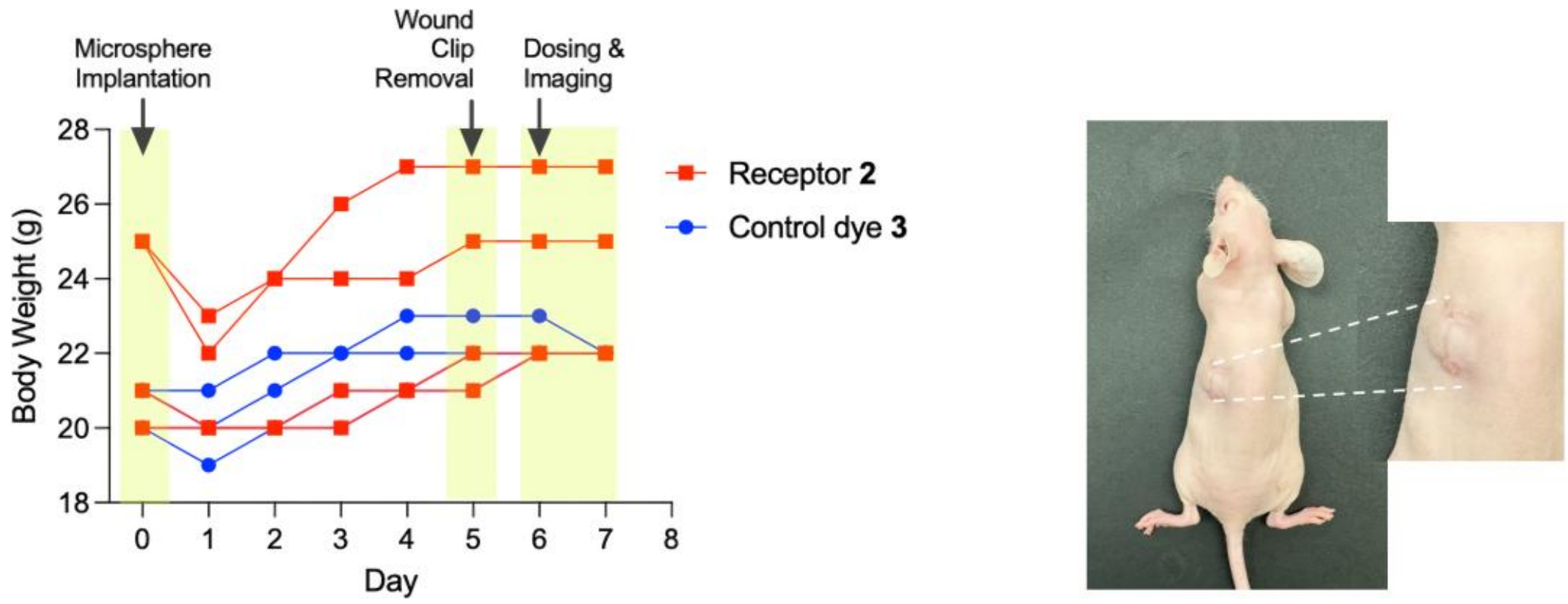

Figure S12. (left) Change in body weight of experimental mice after microsphere implantation, wound clip removal, administration of receptor **2** or control dye **3**, and fluorescence imaging steps. (right) Photograph of a representative nude mouse after wound clip removal but before dosing and imaging.

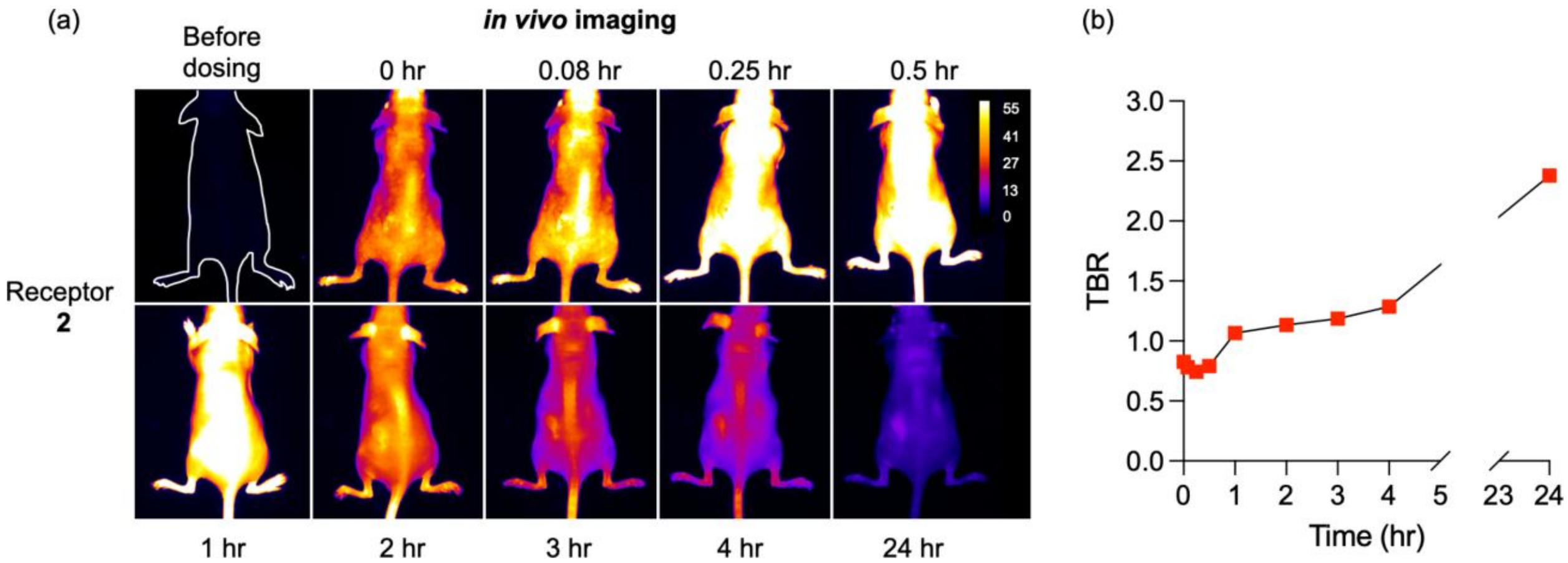

Figure S13. Representative fluorescence images of a living nude mouse with an implanted Ca-crosslinked alginate microsphere; the montage includes multiple early time point images acquired after intravenous dosing with receptor **2** (100 nmol dye/mouse). (b) Plot of Target Background Ratio (TBR) over time after dosing for this single experiment. TBR measured as the ratio of mean pixel intensity (MPI) for microsphere target site divided by MPI for non-target skin site. Fluorescence images acquired using far-red filter; ex: 605/20 nm; em: 670/20 nm; exposure: 3 s; percent power: 50%; F-stop: 2; FOV: 20; binning: low. Fluorescence intensity scale is in arbitrary units.

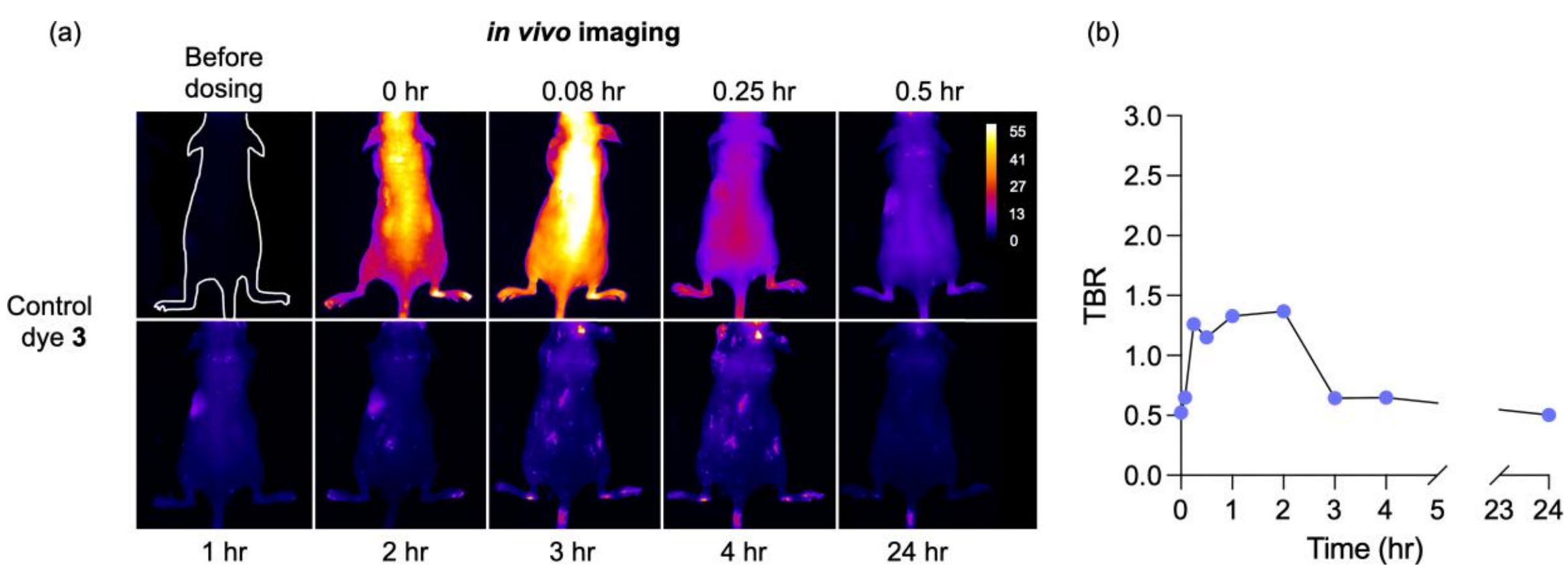

Figure S14. Representative fluorescence images of a living nude mouse with an implanted Ca-crosslinked alginate microsphere; the montage includes multiple early time point images acquired after intravenous dosing with control dye **3** (100 nmol dye/mouse). (b) Plot of Target Background Ratio (TBR) over time after dosing for this single experiment. TBR measured as the ratio of mean pixel intensity (MPI) for microsphere target site divided by MPI for non-target skin site. Fluorescence images acquired using far-red filter; ex: 605/20 nm; em: 670/20 nm; exposure: 3 s; percent power: 50%; F-stop: 2; FOV: 20; binning: low. Fluorescence intensity scale is in arbitrary units.

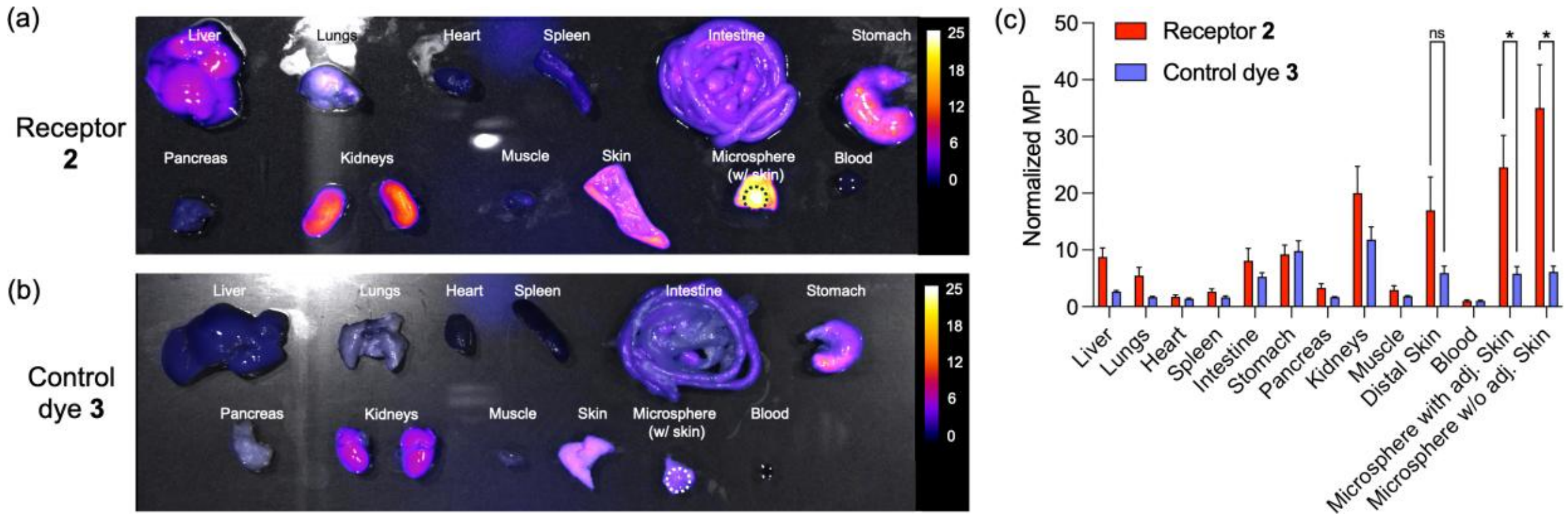

Figure S15. Overlaid brightfield and fluorescence images of harvested organs from two separate nude mice each with an implanted Ca-crosslinked microsphere and sacrificed 24 hours after intravenous dosing with (a) receptor **2** or (b) control dye **3** (100 nmol/mouse). Fluorescence intensity scale is in arbitrary units. Fluorescence image(s) acquired using far-red filter; ex: 605/20 nm; em: 670/20 nm; exposure: 3 s; percent power: 50%; F-stop: 2; FOV: 20; binning: low. (c) Biodistribution analysis of **2** and **3** in nude mice sacrificed 24 hours after intravenous dosing (N = 4) and (N = 3), respectively. The normalized mean pixel intensity (Normalized MPI) is relative to the MPI for the terminal blood sample obtained from the same mouse. Error bars represent ± SEM. * indicates $p < 0.05$ and ‘ns’ (not significant).

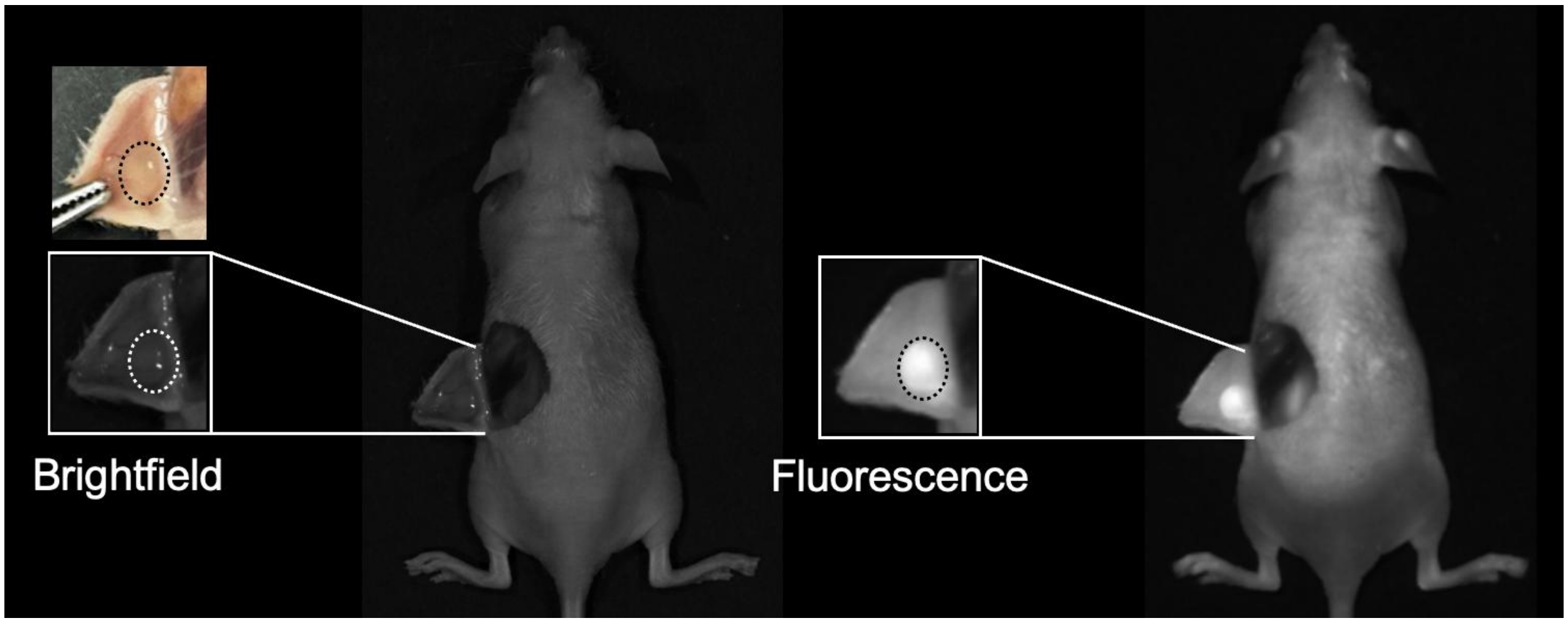

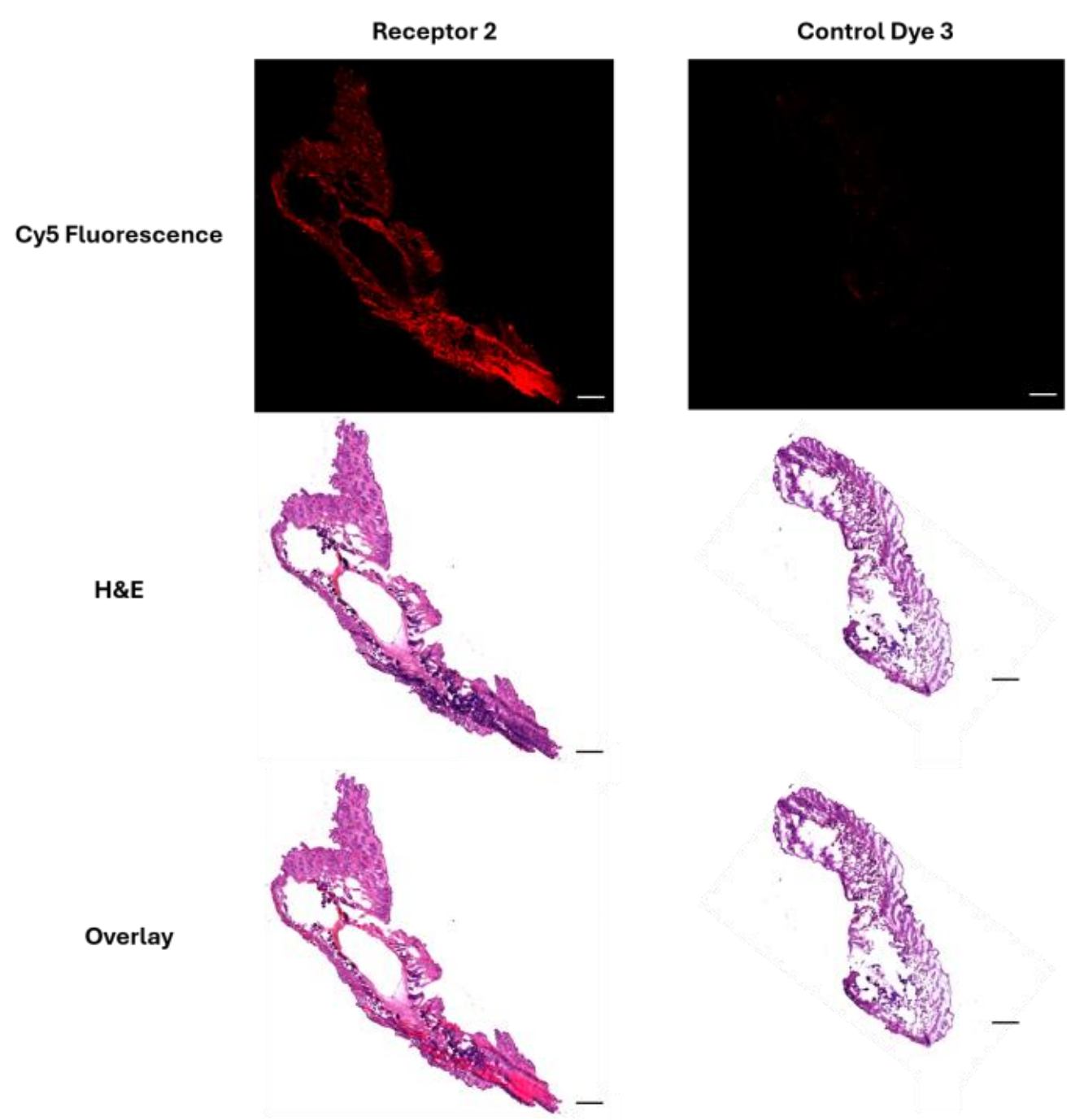

Figure S16. (*top*) Images from a mouse experiment that is a replicate of the one shown in **Figure 6a**. That is, ex vivo deep-red fluorescence (ex: 605 nm, em: 670 nm) and bright field images of the exposed 4 mm diameter microsphere at the subcutaneous implantation site (indicated by dotted oval) in a nude mouse that was dosed intravenously with 100 nmol of receptor **2** and sacrificed 24 hours later. Localization of fluorescent **2** to the implanted alginate is quite apparent. In addition, the color photograph shows that the tissue surrounding the implanted microsphere is not inflamed or infiltrated by immune cells. (*bottom*) Thin sections of tissue that surrounded the excised alginate microsphere. The sections stained with hematoxylin and eosin (H&E) indicate no evidence of infiltrating immune cells. The fluorescent micrographs show that receptor **2** is primarily bound to the implanted alginate. The tissue sample from a mouse with implanted microsphere but dosed with control dye **3** emits very low deep-red fluorescence. Scale bar = 500 µm.

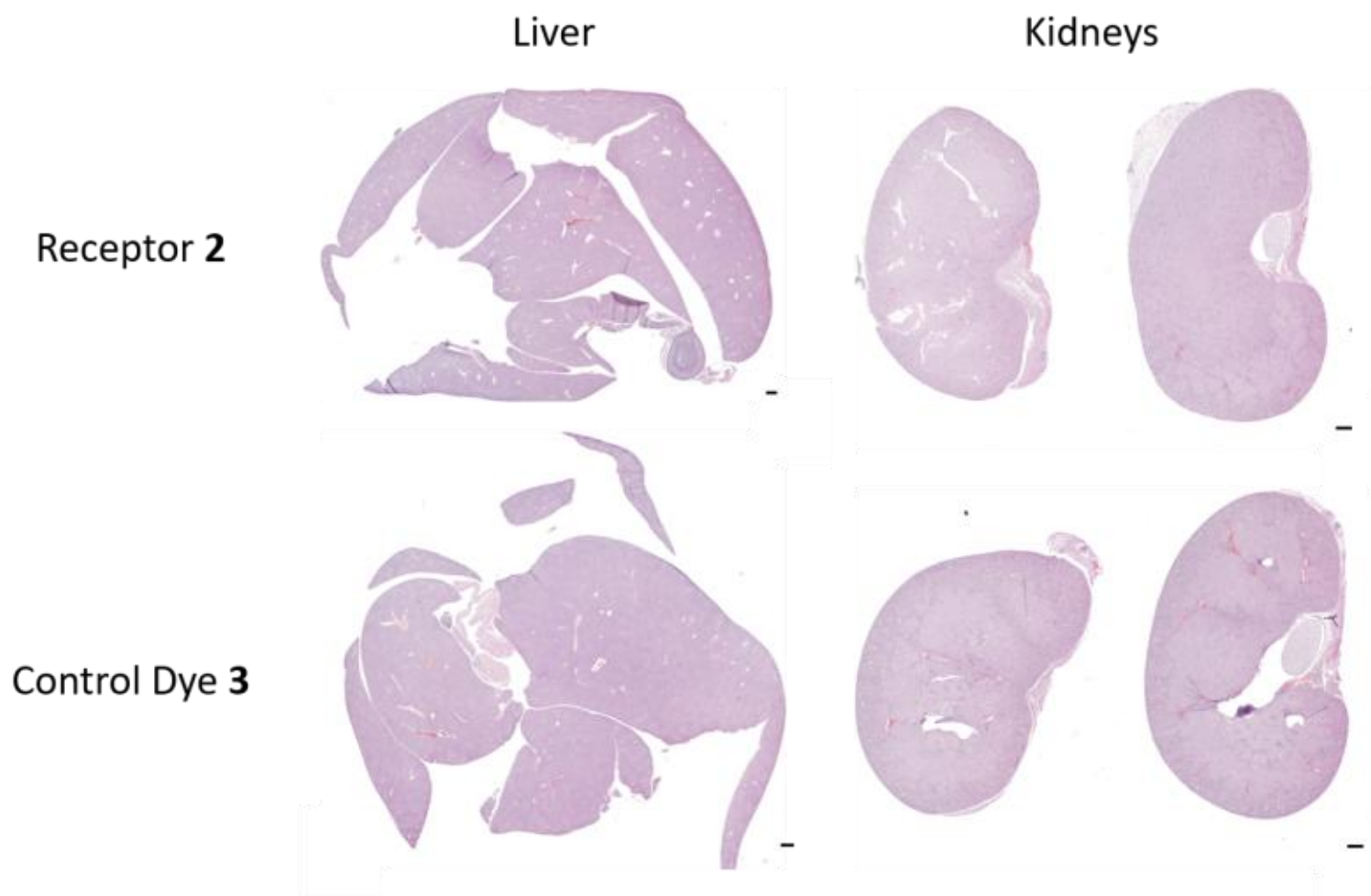

Figure S17. Representative liver and kidney sections from a nude mouse with an implanted Ca-crosslinked alginate microsphere. Six days after microsphere implantation, the mouse was dosed intravenously with 100 nmol of receptor **2** or control dye **3** and sacrificed 24 hours later. The excised organs were embedded in paraffin, sectioned at a thickness of 6 µm and stained with hematoxylin and eosin (H&E). The histological micrographs indicate no morphological abnormalities or signs of tissue damage, which is consistent with the high biocompatibility of unmodified alginate, and the low cell toxicity of **2** and **3 (Figure S11**). Length scale bars = 500 µm.

8. Albumin Binding Measurements.[4]

Aliquots (1 µL) of (a) receptor **2** or (b) control dye **3** (from 1 mM stock solutions) were added to 1 mL of bovine serum albumin (BSA) (500 µM) in phosphate buffer saline at room temperature. The BSA tryptophan fluorescence intensity (excitation: 280 nm; slit width: 3 nm) was plotted as a function of the dye concentration to obtain the slope which equaled the apparent association constant K_{app} ± SD (M^{-1}).

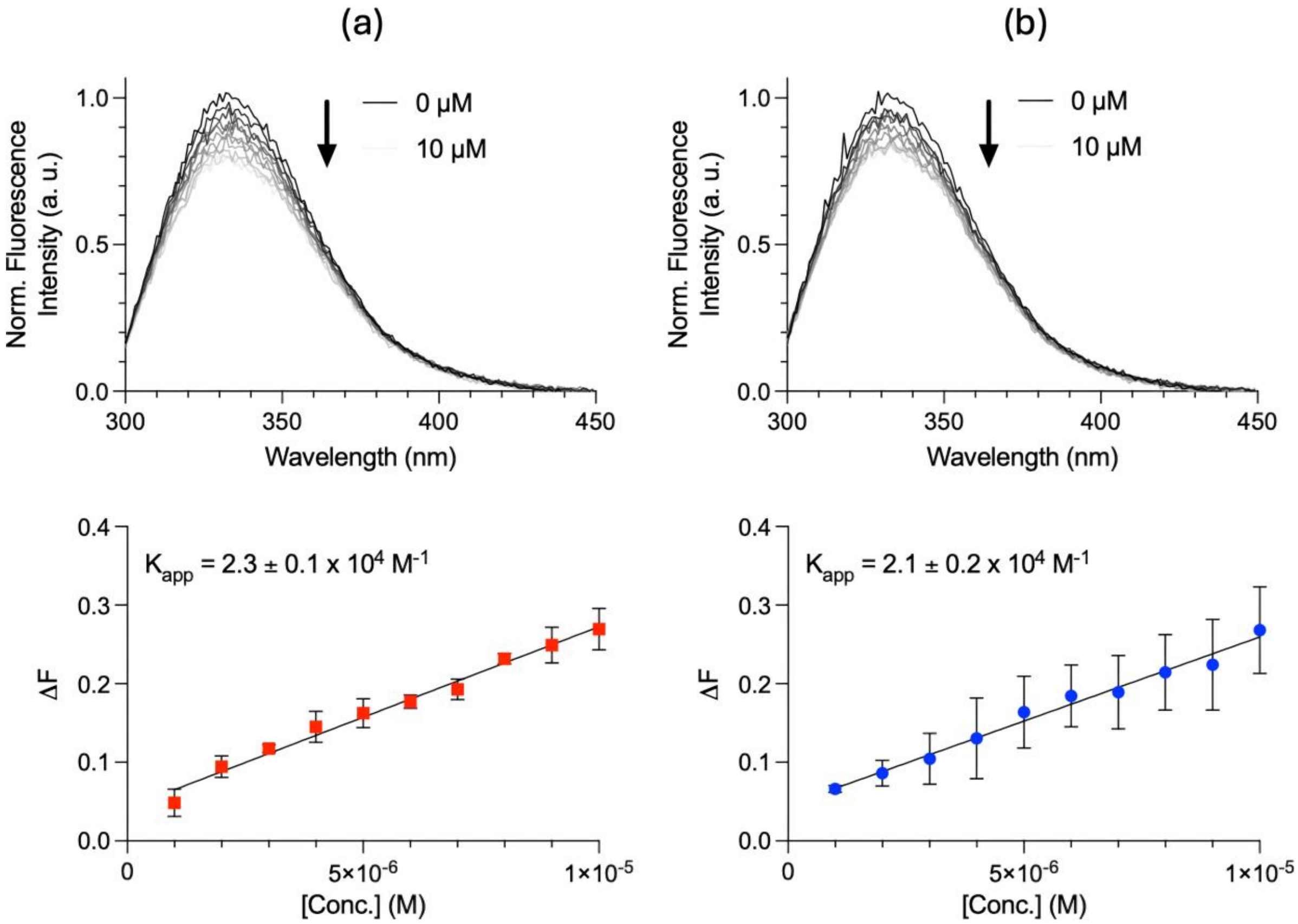

Figure S18. BSA tryptophan fluorescence upon incremental addition of (a) receptor **2** or (b) control dye **3**.

Conclusion of Albumin Binding Measurements: The measured values of K_{app} for **2** or **3** binding BSA are twenty times lower than the values determined (using the same analytical method) for other dyes that are considered strong binders of albumin. One example is the following:[4]

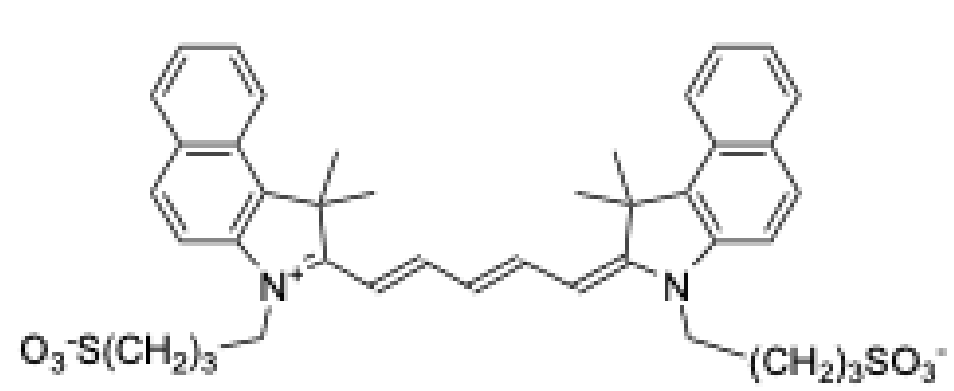

ICB: K_{app} = 4.2 x 10^5 M^{-1}

9. References

(1) H. Fuchida, S. Tabata, N. Shindo, I. Takashima, Q. Leng, Y. Hatsuyama, I. Hamachi and A. Akio Ojida, *B. Chem. Soc. Jpn.*, 2015, **88**, 784-791, DOI: 10.1246/bcsj.20150037.

(2) R. Nanjunda, E. A. Owens, L. Mickelson, S. Alyabyey, N. Kilpatrick, S. Wang, M. Henary and W. D. Wilson, *Bioorg. Med. Chem.*, 2012, **20**, 7002-7011, DOI: 10.1016/j.bmc.2012.10.008.

(3) J. J. Morsby, Z. Zhang, A. Burchett, M. Datta and B. D. Smith, *Chem. Sci.*, 2024, **15**, 3633-3639, DOI: 10.1039/d3sc06058f.

(4) R. S. Gamage and B. D. Smith, *Chem. Biomed. Imaging.*, 2024, **2**, 384-397, DOI: 10.1021/cbmi.4c00008.
